# Preference reversals in ethicality judgments of medical treatments

**DOI:** 10.1371/journal.pone.0319233

**Published:** 2025-04-29

**Authors:** Benjamin A. Lemli, Justin F. Landy

**Affiliations:** 1 Department of Psychology and Neuroscience, Nova Southeastern University, Fort Lauderdale, Florida, United States of America,; 2 Department of Psychology, Miami University, Oxford, Ohio, United States; Queen Mary University of London School of Business and Management, UNITED KINGDOM OF GREAT BRITAIN AND NORTHERN IRELAND

## Abstract

In medical ethics, there is often a tradeoff between maximizing treatment efficacy and alleviating patient suffering. We adapt methods from consumer behavior research to examine whether ethicality judgments of medical treatments that vary on these dimensions exhibit preference reversals across tasks and evaluation modes. Specifically, we present participants with pairs of treatments that symmetrically dominate one another: one is more effective, while the other improves patients’ quality-of-life. Across three studies (total *N* = 500), we demonstrate classic preference reversals in lay medical ethics judgments: participants prioritized efficacy over quality-of-life concerns in matching tasks more than choice and rating tasks, in between-subjects (Study 1) and within-subjects (Study 2) designs, and in joint evaluation more than sequential evaluation (Study 3). We interpret these findings in light of previous research on preference reversals in other domains and discuss implications for healthcare and moral psychology.

## Introduction

Patients with medical ailments often face a wide array of treatment options that vary on multiple dimensions. One tradeoff that often presents itself in medical care is between maximizing a patient’s odds of survival (i.e., treatment efficacy) and minimizing the patient’s suffering (i.e., quality-of-life considerations; see, e.g., [1]). For any individual patient, how they prioritize these two concerns is a matter of personal preference, not ethics. But practitioners, administrators, and policymakers must also make decisions about what treatments to recommend to patients, and how to allocate funding. Indeed, over half of people with cancer report struggling to pay their medical bills and meet their cost of living [2–4]. As such, financial aid and free treatment programs are a part of how Americans receive cancer care and make medical decisions more generally. Should money be put toward a very painful treatment that is marginally more effective than a less painful one, prioritizing efficacy at all costs? Should quality-of-life considerations be factored in? How much must the odds of survival increase to make additional patient suffering “worth it”? Such questions raise undeniably ethical considerations.

These sorts of questions remain unresolved in bioethics and applied medical ethics [5–7]. For our purposes, complex ethical questions like this provide useful and theoretically interesting opportunities to study judgment and decision making in the ethical domain, because people could employ a variety of strategies to answer them. In the context of care for cancer patients, one might, for instance, evaluate different treatment programs on various dimensions in isolation, or evaluate programs competing for funding side-by-side. One might employ some kind of subjective rating of how ethical each program is, or choose between competing pairs of programs, or even try to answer the above question about when additional suffering is “worth it” directly by setting a threshold – if the odds of survival from a treatment are *this much* higher than an alternative, that morally justifies the patient’s reduced quality of life during treatment. All of these strategies are normatively equivalent, in the sense that employing different strategies does not change anything about the treatments being assessed. Therefore, if people hold consistent preferences and priorities while assessing options, they should all lead to the same ethical conclusions. Indeed, in clinical settings, it is generally assumed that patients and medical professionals judge the ethicality of available treatment options by assessing their outcomes, with the option that helps the most and hurts the least being the most ethical [8]. This would imply that people should make consistent judgments regardless of the strategy they employ. In the present research, we demonstrate that this is not always the case.

While much attention has been paid to elucidating domain-specific cognitive processes that drive moral judgments, less attention has been paid to domain-general influences of judgmental tasks on ethics judgments. Both the fields of moral psychology and medical decision making tend to regard preferences as stable, in that they may be susceptible to biases or heuristics, but they are at least relatively consistent across normatively equivalent tasks. Within moral psychology, research on interpersonal differences [9,10], context and framing [11], attention and memory [12–14], Moral Grammar [15–17], Social Learning [18–20], Moral Intuition [21–23], computational decision making [24–27]), and behavioral neuroscience [28–30] generally assume (at least implicitly) that a judgment is independent of the task used to elicit it.

In the present research, we demonstrate that three preference reversals that have been shown to characterize consumer preferences also manifest in moral judgments about medical treatments. We thus build on a long tradition of examining human moral judgment in the medical domain. This approach arguably dates to Kohlberg’s famous “Heinz dilemma” [31] and is exemplified by more recent stimuli like the “organ donation dilemma” [32–34] and the “hospital administrator dilemma” [35,36].

### Preference reversals

For the purposes of this research, we define a *preference* as a judgment of the superiority of one option over another on some evaluative dimension (in this case, ethicality). A *preference reversal* (PR) occurs when one option is preferred to (i.e., judged superior to) a second in one method, but the second option is preferred to the first in another, normatively equivalent method. Because it is logically impossible for one option to be both superior and inferior to another on the same evaluative dimension, PRs constitute a clear bias and deviation from rationality.

Economists and consumer behavior researchers long assumed that people’s preferences for one option over another should be stable because they reflect some calculation of utility [37–40]. This assumption was challenged by Slovic and Lichtenstein’s [40–43] famous gambling studies, in which participants chose one gamble over another, but priced the gambles in a way that implied the opposite choice. Following these seminal studies, research has demonstrated that different elicitation tasks [44], evaluation modes [45], accompanying options [46], and framings [47] predictably elicit different judgment strategies, and therefore consistently produce PRs, where one option is preferred in one framing, evaluation mode, or task, but an alternative is preferred in a different, normatively equivalent, framing, evaluation mode, or task. In the present article, we focus on evaluation mode and elicitation task. We chose to manipulate elicitation task because it is the oldest and most common PR effect in the psychological and economic literature [40], and evaluation mode because of the growing body of literature demonstrating effects of evaluation mode in various domains [e.g., 48–51]. Moreover, recent research has examined PRs across evaluation mode and elicitation task in the moral domain [52]. Evaluation mode refers to whether stimuli are evaluated separately (only one option is evaluated), sequentially (options are presented one at a time until all have been evaluated), or jointly (options are evaluated in pairs). Elicitation task refers to the response scale or task given to an individual to express their preference.

### Choice, rating, and matching

In research on PRs, three elicitation tasks that are commonly used are *choice, rating,* and *matching.* All three tasks are typically used to elicit a preference between two options, each characterized by two attributes [44,53–59]. In the standard research design, one option is superior in regard to one attribute (e.g., price), while the second is superior in regard to the other (e.g., quality, brand reputation, etc.). Choice tasks require participants to select which of the two options they prefer. Rating tasks ask for continuous ratings, usually on a Likert-type scale, of the desirability of each option, the likelihood of purchasing each option, or some similar operationalization of preference. Matching tasks leave one quantitative attribute of one option blank and ask participants to indicate what value that attribute would have to have to make them indifferent between the two options. A preference is inferred from this judgment by assessing whether the participant’s value is inferior or superior to the value of the attribute in the choice or rating task [44].

In two-option, two-attribute tasks of this kind, participants often indicate a preference for one option in choice, but indicate a preference for the alternative in a matching task [44,53–59]. Similarly, participants often *rate* one option as superior, but indicate a preference for the other option in matching [60,61]. More specifically, quantitative features of options tend to be more influential in matching tasks, because matching forces decision makers to make effortful, inter-option tradeoffs. Conversely, choice and rating tasks tend to elicit a focus on qualitative information, particularly affectively-charged or highly salient attributes, and may cause decision makers to rely on global assessments of attributes and options [44,55,62–64]. In summary, in choice and rating, participants often express a preference for the option that is superior on an affective, qualitative dimension, but in matching, they tend to express a preference for the option that is superior on a quantitative dimension that requires deliberative processing to assess.

Tversky et al. [44] demonstrated choice-matching PRs, and proposed what is still the most popular explanation for such reversals, the Prominence Hypothesis: the more “prominent” (i.e., important) attribute of two options will weigh more heavily in choice than in matching. In Tversky et al.’s work, and other early choice-versus-matching research, options were gambles, consumer products, or outcomes like the implementation of a public policy. Prominent attributes were qualitative things like the brand or quality of a product, and “non-prominent” attributes were almost always quantitative things like prices or consumer ratings [44,53,55,56,58,65–69].

Recently, a series of studies examined PRs between choice, rating, and matching in the moral domain [52]. Participants rated the moral rightness of two different “sacrificial harms”, in which a person kills one person to save a larger number of people. Following the standard methodology described above, they presented participants with pairs of scenarios that varied on two attributes: the heinousness of the violent action (a qualitative, affective attribute) and the number of lives saved (a quantitative attribute). Adapting the standard methodology, in which each action dominated the other in regard to one attribute, they documented PRs, such that the relative judged moral rightness of the two sacrificial harms reversed between matching and choice, and between matching and rating. Specifically, they found that participants said that the more heinous action that saved more lives was more moral than the less heinous action that saved fewer lives in matching much more often than they did in choice and rating. We adopt a similar method in the present research, in the domain of medical ethics.

Choice-matching PRs have also recently been examined in the medical domain [61,68,70–73]. Rooted in the Prominence Hypothesis, these studies pit a clearly prominent attribute (e.g., the quality of care, an identifiable victim, medical outcomes) against a clearly less-prominent attribute (i.e., cost). Which attribute should be considered more “prominent” (i.e., important) in dilemmas like ours where treatment efficacy (i.e., rates of survival) is pitted against quality-of-life factors like unpleasant side effects, is not nearly so clear. An argument could be made (and sometimes is, see [45,52,63,70]) that attributes that evoke strong affect, require no comparison or calculation to utilize in a decision, and are evaluable without a comparison item (e.g., painful symptoms experienced during a medical treatment) are more prominent than those that lack these features (e.g., numerical treatment efficacy), though one could contrastingly argue that treatment efficacy sometimes presents a more salient feature in assessment of medical treatments than alleviation of symptoms.

The research of Erlandsson and colleagues is of particular relevance to the present work because it illustrates effects that are, by definition, restricted to the moral domain, like the identifiable victim effect, are not immune to task-induced PRs [48,70]. In one study [73], participants were presented with medical treatment dilemmas describing two groups of people in need. The outcomes for one group were superior on the quantitative attribute of lives saved, but the other was an in-group, or a social group that participants might otherwise prefer to help (e.g., children). When participants were forced to choose between the programs they had *matched* as equally attractive, they *chose* programs that helped a demographic group the participants preferred. However, this method leaves open the possibility that this systematic pattern of judgment only reflects a “tiebreaking” procedure, rather than a genuine inconsistency (see [44]). We employ a different method, in which both attributes are fixed in the choice task and preferences in the matching task are inferred by comparison to these fixed values, avoiding this concern. Moreover, this prior work pitted the number of lives saved against demographic information. While the former is clearly a normatively relevant consideration in medical ethics, the latter presents a stimulus that is not universally normatively significant, and is interpersonally variant in terms of not only strength, but also valence as a judgmental stimulus [48]. Some ethical systems might, for instance, elicit greater ethical consideration for ingroup members, or individuals of certain social statuses (e.g., children) than outgroup members or individuals of other social statuses (e.g., adult), while others may not. So, in the present research, we pit two normatively relevant attributes (treatment efficacy and quality of life) against one another.

Some research finds PRs between choice and rating [69,74], but the majority of research indicates choice and rating both tend to lead to preferences for options that are superior on a qualitative attribute, while matching leads to preferences for options superior on a quantitative attribute that requires some degree of comparison and calculation to assess [52,66,75–77]. Additionally, research showing PRs between choice and rating typically confounds elicitation task with evaluation mode, with choice tasks being presented in joint evaluation, and rating tasks presented in separate or sequential evaluation (e.g., [67,78–80]). As we detail below, joint evaluation tends to evoke more deliberative thinking, while separate and sequential evaluation tend to evoke more intuitive, heuristic thinking. So, PRs between choice and rating – when they occur at all – may be explained by presentation mode, rather than by elicitation task. We therefore did not expect to find any consistent PRs between choice and rating.

### Joint, separate, and sequential evaluation

Another task-related manipulation that has been shown to produce PRs between consumer products is whether the products are evaluated one at a time (separate or sequential evaluation) or two at a time, side-by-side (joint evaluation). Hsee [45] demonstrated that this effect is due to the differential *evaluability* of attributes. Some attributes of products are easier to evaluate in separate evaluation, and some are only easily evaluated when compared to a similar product with an analogous attribute.

The evaluability effect has been repeatedly demonstrated in consumer behavior research in both sequential-versus-joint evaluation and separate-versus-joint evaluation designs, and is typically provided as an explanation for these specific types of PR (e.g., [45,49,81–87]). Much like the research on different elicitation tasks reviewed above, the attribute that is easily evaluated in isolation is often qualitative in nature, while the attribute that requires joint presentation to be evaluated is almost always quantitative. Similar to matching tasks (versus choice and rating tasks), joint evaluation has been suggested to elicit more deliberative cognition than separate or sequential evaluation. Specifically, joint evaluation has been shown to take more time [88–92], promote greater inter-attribute comparison [74,81,87,93], and produce less affect-based and more quantitative-based reasoning [94,95] than separate and sequential evaluation. Notably, if evaluability is indeed the reason for observed PRs between separate or sequential versus joint evaluation, it seems likely that such effects would be weaker (rather than stronger) when comparing joint evaluation with sequential (rather than separate), as repeated presentations of single options provide some points of comparison, making even sequential options more evaluable. Thus, our use of sequential (rather than separate) evaluation is likely conservative; if anything, it would bias *against* observing PRs, compared to using separate evaluation.

The evaluability effect has rarely been examined in the domain of medicine. One study utilized sequential and joint evaluation to show that participants made judgments about the desirability of choosing specific health-care providers based on the evaluability of the available information [50]. In sequential evaluation, attributes that are easy to evaluate in isolation, such as travel distance, predicted participants’ judgments better than attributes that were harder to evaluate in isolation. Conversely, criteria that were hard to evaluate in isolation, such as the success rate of a procedure, weighed more heavily in joint evaluation. However, unlike our work, this study was not concerned with ethical judgments of treatments, but rather with personal preferences for where to seek out medical care for oneself.

The present research examines ethical judgments in the domain of medicine. The only prior work like this that manipulates evaluation mode was conducted by Erlandsson [48] who presented a large-scale research project that investigated several effects that lead to a reversal of preference across evaluation modes in the context of helping dilemmas. In Erlandsson’s dilemmas, multiple groups are in dire need of life-saving help, but limited resources require participants to allocate help to some demographic groups over others. In sequential evaluation, participants rated programs that helped different groups similarly, regardless of the number of people they helped. In contrast, attractiveness ratings for programs evaluated jointly were largely influenced by the quantitative attribute of number of lives saved (with the more effective programs being rated as superior), rather than the qualitative attribute of what demographic category was helped. In the present research, we pit treatment efficacy against another (arguably) morally relevant factor, patient suffering, rather than a normatively irrelevant factor, patient demographics.

### The present research

We investigate preference reversals in ethical judgments of medical treatment programs elicited in forced choice, rating, and matching tasks, as well as joint evaluation (JE) and sequential evaluation (SE). Our stimuli required participants to make third-party judgments of ethicality about decisions to fund various programs to treat patients with cancer. These stimuli pit hypothetical patients’ chances of survival against their quality of life, such that some treatment options have superior survival outcomes, but others more effectively alleviate patients’ suffering. We find that ethical preferences between such programs depend on how they are elicited and therefore can only be interpreted relative to their evaluation mode and elicitation task.

The present studies utilize medical treatments and judgments of ethicality for several reasons. First, our stimuli enable us to examine moral preferences in an applied, naturalistic domain. This is not to say that our stimuli and experimental measures have high external validity, but they do at least bear a resemblance to real tradeoffs faced by patients, practitioners, and policymakers. Indeed, tradeoffs between quality of life and longevity really do occur in cancer treatment [1,5–7]. Second, the present research builds on a small but growing body of research on PRs in medical decision making (e.g., [50,57,96,97]), and extends research on PRs in moral judgment [52] to medical ethics. Third, our stimuli allow us to carefully control two competing attributes of a treatment as they relate to its ethical status: its efficacy, and the presence of painful symptoms during treatment. Thus, we adapt the two-option, two-attribute structure of classic research on PRs to the study of lay medical ethics.

Another reason to present participants with a task regarding an ethical judgment is to avoid confounding value with desirability. Some scholars have argued that task effects are not caused by a true reversal in preference or belief, but by a difference in the nature of the elicited judgment between tasks [94,98–101]. From this perspective, it has been argued that some tasks including rating reflect an affectual attitude, while choice tasks reflect a calculation of preferential ordering of options, and matching tasks reflect an assessment of (monetary) value [95]. In this case it would be possible for an individual to judge a product as more valuable than an alternative, and also prefer to own or purchase the alternative. In such a case, a matching task task implicitly asks the question, “what is this worth?”, while a willingness to buy rating, or a choice asks the question, “what do you prefer?”.

However, while traditional preferential tasks may elicit judgments of value or preference, depending on the nature of the task, the present study’s tasks address efficacy (number of individuals who survive). It might be consistent to say a product could cost more than an alternative one prefers, but it does not seem consistent to say that an individual both thinks that an end needs to meet certain criteria to ethically justify some means, but an alternative that does not meet said criteria is ethically justified. In judgments of ethicality, it seems reasonable to assume that rating, choice, and matching are fundamentally eliciting the same judgment: “Is it ethically preferable to save the most lives, or to prevent the suffering of patients?” Further, while products, services, and even public policies may already be associated with some monetary value or utilitarian worth, for most people, a given number of lives saved has no standard convertible value. The attribute, number of lives saved, draws clear attention to the moral features of the stimuli, and does not rely on any previous knowledge about quantitative worth of the stimuli.

Studies 1a and 1b investigated the effect of matching, rating, and choice tasks on preferences utilizing a between-participants design. We predicted that:

H1) The higher-efficacy/symptom-present programs will be judged as more ethical to fund than the lower-efficacy/symptom-eliminated programs at a significantly higher rate in the matching condition than the choice and rating conditions.

Furthermore, we also predicted, based on prior findings [52,63,66,74,75], that:

H2a) Choice and rating tasks would not elicit significantly different rates of ethical preference for the higher-efficacy/symptom-present over the lower-efficacy/symptom-eliminated programs.

Or, conversely, we investigated in Study 1b if:

H2b) Participants would prefer the higher-efficacy/symptom-present programs over the lower-efficacy/symptom-eliminated programs more often in choice than rating.

Study 2 employed a multi-session, within-subjects design, in which participants completed a matching task and either a rating or choice task roughly two weeks apart, as well as individual difference measures of analytic thinking, dispositional thinking style, personality, and moral identity. Based on the results of Study 1, we predicted that we would again find support for H1 and H2a, this time within-subjects. We also explored whether individual differences in susceptibility to PRs are predictable from our individual difference measures, but we did not formulate any specific hypotheses about this.

In Study 3, all participants completed a rating task in which they evaluated treatment programs either jointly or sequentially. In agreement with the evaluability hypothesis, we predicted that:

H3) When symptoms are the result of omission of action (i.e., failure to implement a symptom-alleviating treatment program), programs with higher efficacy but painful symptoms will be rated as more ethical in joint evaluation than sequential evaluation because efficacy is easier to evaluate in comparison, while symptoms will be more salient when assessing individual treatment programs.

## Ethics and open practices

All studies were approved by the Institutional Review Board at Nova Southeastern University. All studies were pre-registered, and all materials, data, and analysis scripts are publicly available athttps://osf.io/w6j48. We report how we determined our sample sizes, all data exclusions, all manipulations, and all measures in all studies.

## Pretests

Two pretests (*N* = 63; 62) were conducted to develop the final set of stimuli (see S1 Text File and S1 and S2 Tables in the Supporting Information for full methodology, rationale, and results). Both pretests sampled participants over the age of 18 located in the US via Amazon’s Mechanical Turk (“MTurk”) and required them to rate how ethical treatment programs were to implement in a medical context. The first pretest tested which symptoms constituted an effective manipulation, in that they produced a preference for an option with a lower efficacy that alleviated a symptom over an option that had a higher efficacy, but did not alleviate that symptom, in sequential evaluation. Based on this pretest, we retained nine symptoms for our final stimuli. The second pretest tested which range of efficacies between programs produced an effective manipulation in joint evaluation by producing a preference for a program that had higher efficacy, but did not alleviate a symptom, compared to a lower-efficacy program that did. The second pretest examined two overall ranges for all program efficacies, one between 40% and 52%, and one between 78% and 90%. On the basis of this pretest, the second, lower efficacy range was selected for the final stimuli. Thus, based on our pretests, we developed nine pairs of treatment programs that symmetrically dominate one another: one saves a greater percentage of patients’ lives, while the other improves patients’ quality-of-life by eliminating an unpleasant symptom.

### Studies 1a and 1b: Choice and rating versus matching, between-participants

In Studies 1a and 1b, participants were asked to evaluate how ethical it was to fund each medical program by rating, choosing between, or matching the ethicality of programs. In study 1a, we predicted that the higher-efficacy/symptom-present programs would be judged as more ethical to fund at a significantly higher rate in the matching condition than the choice and rating conditions (H1), and that forced-choice and rating tasks would not elicit significantly different rates of ethical preference for the higher-efficacy/symptom-present and low-efficacy-symptom-eliminated programs (H2a). In study 1b, we retained H1, but increased the number of participants in the choice and rating conditions to examine a potential difference between rating and choice (H2b), such that participants would prefer the higher-efficacy/symptom-present programs over the lower-efficacy/symptom-eliminated programs more often in choice than rating.

Studies 1a and 1b were pre-registered at https://aspredicted.org/MYY_Q2Z and https://aspredicted.org/RZK_4LJ, respectively.

### Method

#### Participants.

Participants in Studies 1a and 1b were adults located in the United States, recruited via MTurk. Participants provided consent via a computerized form and received a written debrief. This informed consent procedure was approved by the Nova Southeastern University Institutional Review Board. One hundred-fifty participants completed Study 1a on November 15^th^, 2022, and one hundred-one participants completed Study 1b on November 28th, 2022, however data from 38 participants in Study 1a and 64 participants in Study 1b were excluded due to failed comprehension checks, leaving final samples of *N* = 63 in Study 1a and *N* = 86 in Study 1

#### Materials. 

**Fig 1:**
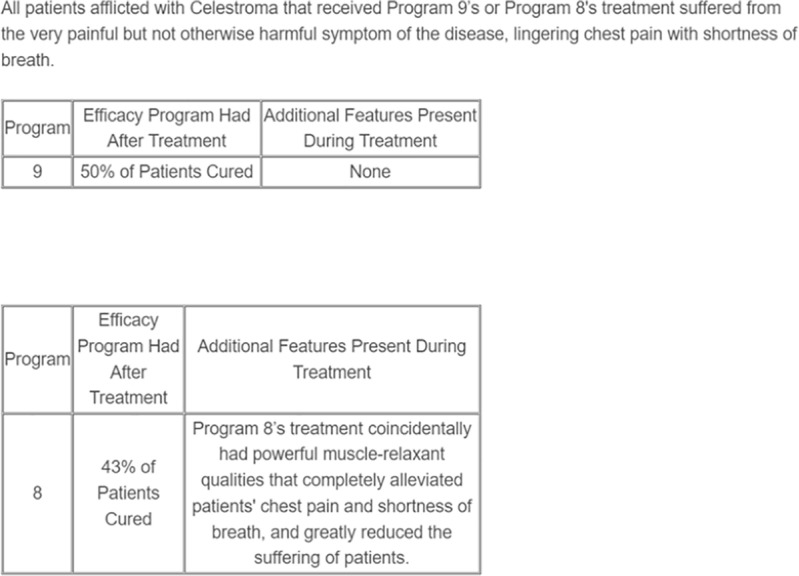
Example stimulus, Study 1.

Stimuli in Studies 1a and 1b consisted of eighteen treatment programs (nine pairs) developed in the pretests, presented in tables displaying the efficacy and symptom information for each program. Above each pair of programs was a short block of text indicating that all patients who received treatment suffered from Celestroma (a fictitious cancer; see S2 Table and S1–S16 Figs in the Supporting Information for full materials) and experienced a particular symptom of the illness (tendonitis, ocular migraine, chronic depression, lingering chest pain and shortness of breath, eczema, onycholysis, abdominal pain, arthralgia, or painful sores in and around the mouth) during treatment. All patients that received either program would have otherwise experienced the symptom during treatment (see Fig 1 for an example stimulus).

#### Procedure.

After providing informed consent, participants in Studiess 1a and 1b were told that they were about to see several treatment programs that were implemented at similar hospitals to treat terminally ill patients with Celestroma. Participants were instructed to evaluate how ethical it was to implement each program, considering that funding is often limited, and real-life decisions about who to help and how to help them are faced by clinicians and policymakers with limited resources every day. Participants were randomly assigned one of three conditions and presented with the program-pairs on separate pages. In the rating condition, participants rated how ethical it was to choose each program for funding on a nine-point Likert-type scale ranging from −4 (very unethical) to +4 (very ethical). Participants in the choice condition indicated which of the two programs was more ethical to choose for funding. In the matching condition, the efficacy for one program in each pair was missing, and participants were instructed to indicate what efficacy that program would be required to have to make both programs equally ethical to fund.

Before being presented with the focal stimuli, participants were given a comprehension check described as a “warm up question”. Rather than forcing participants to make tradeoffs between opposing attributes, the comprehension check featured two programs, one of which dominated the other on both attributes. Specifically, the comprehension check item presented a program that had 78% efficacy and eliminated a symptom, and a program with 40% efficacy that did not eliminate the symptom. Because one of the programs in the comprehension check both saved more lives and eliminated a symptom experienced by patients in the other program, participants that indicated that the inferior program was more ethical than the superior program apparently did not understand the task or were not paying attention. Following our pre-registration, these participants were excluded from analysis.

In Study 1a, half of all participants completed the matching condition, while a quarter of participants completed either the rating or choice condition. In Study 1b, an equal number of participants were assigned each task condition to provide better power to compare the choice and rating conditions. Complete separate analyses for Studies 1a and 1b can be found in the Supporting Information in S3 Table and S2 Text file; for the sake of brevity and to maximize statistical power, we present combined analyses below. Treatment programs were counterbalanced for presentation order and which program had a missing value for efficacy in the matching condition. After evaluating all of the programs, participants completed a brief demographic survey and were debriefed and compensated $1 for their participation.

#### Analysis plan.

The analysis procedures of Studies 1a and 1b were identical. The dependent variable in the choice condition was the proportion of program-pairs in which the higher-efficacy/symptom-present program was chosen as more ethical to fund and implement. The dependent variable in the rating condition was the proportion of program-pairs in which the higher-efficacy/symptom-present program was rated as more ethical to fund and implement. The dependent variable in the matching condition was calculated differently depending on whether the matched attribute (efficacy) was missing from the higher- or lower-efficacy program. When the higher-efficacy/symptom-present program’s efficacy was missing, the dependent variable was calculated as the proportion of program-pairs in which the participant-matched value is less than the value in the choice and rating conditions. When the lower-efficacy/symptom-eliminated program’s efficacy was missing, the dependent variable was calculated as the proportion of program-pairs in which the participant-matched value is greater than the value in the choice and rating conditions.

Across all conditions, responses to program-pairs that indicated a preference for the higher-efficacy/symptom-present program were coded as 1, and responses that indicated a preference for the lower-efficacy/symptom-eliminated program were coded as 0. Responses in the matching condition that were identical to the values of the programs in the choice and rating conditions, and responses in the rating condition in which both programs were rated as equally ethical indicate indifference and were excluded from analysis in order to produce meaningful proportions of response-types, following the analyses of classic consumer PR studies (see [44,53,66,78]). We made this choice to demonstrate PR effects with similar methods and identical analyses to the studies that originally demonstrated them, and because our interest is in ethical *preferences –* if a participant does not express a preference between options in a given scenario-pair, then that data point is not meaningful in these analyses. Thus, the final dependent variable represents the proportion of trials in which a participant expressed a preference for the higher-efficacy/symptom-present program, excluding trials in which they expressed indifference. Because Studies 1a and 1b were identical, with the exception of the condition cell sizes, we combined the results from both studies to maximize statistical power (separate analyses of Studies 1a and 1b before and after exclusions are presented in the Supporting Information in S3 and S4 Tables).

### Results

There were significantly more ties in the rating condition than the matching condition because participants were able to easily equate options without having to know what the missing value of the matched attribute for a given program in the analogous condition was, *t*(143) = 11.97, *p* <.001, *d* = 1.0. Simply put, it was much easier to express indifference in rating than matching, and impossible to express indifference in choice. This pattern is inherent to the tasks in the present study, and it would be uninformative to conduct further analysis on the rate of indifferent responses across conditions.

Consistent with H2a, the choice (*M* =.57, *SD* =.34) and rating (*M* =.62, *SD* =.27) conditions did not significantly differ, *t*(100) = 0.81, *p* =.419, *d* = 0.16 (See S5 and S6 Tables in the Supporting Information for separate analyses of choice and rating conditions by item). We followed up on this null-hypothesis test by conducting a Two One-Sided Tests (TOST) equivalence test [102] using the TOSTER package for R [103]. This analysis was not pre-registered, but sheds light on whether the two conditions can be considered statistically equivalent to one another. Following the recommendation of Lakens [103], we used the G*Power software package [104] to conduct a sensitivity analysis to determine the smallest effect size of interest [103] implied by our final sample size after exclusions. We determined that we had 80% power to detect an effect size of *d* = 0.56, so we set the equivalence bounds in the TOST at ±0.56. The TOST was significant, *t*(100) = 2.01, *p* =.024, so we reject the null hypothesis that there is a true mean difference larger than the SESOI. So, while we cannot rule out a true effect of small-to-medium size (Cohen, 1988), we can say with confidence that it is highly unlikely that there is a true effect large enough to be reliably detected in our sample. Notably, the observed effect size, *d* = 0.16 is far smaller than the SESOI, is small in an absolute sense, and, based on an a priori power analysis using G*Power, would require a very large sample size of *N* = 1230 to be detectable with 80% power. Thus, looking across the null hypothesis tests, TOSTs, and observed effect sizes, we are confident that any true difference is likely to be negligible, and preferences in the choice and rating conditions are meaningfully similar. Based on these results, we combined the choice and rating conditions, following our pre-registrations.

Consistent with H1, participants in the matching condition showed stronger preferences for the higher-efficacy/ symptom-present program (*M* =.85, *SD* =.32) than participants in the combined choice/rating condition (*M* =.60, *SD* =.31), *t*(145) = 4.56, *p* <.001, *d* = 0.75. Two-proportion z-tests for each scenario-pair showed that participants responded that the higher-efficacy/symptom-present program was more ethical to fund significantly more often in matching than the combined choice and rating conditions for every program pair (see Table 1).

**Table 1:** Proportions of participants indicating the higher-efficacy/symptom-present program is more ethical, and total number of participant responses, in Study 1, by stimulus pair and condition.

Program Pair	Choice/Rating	Matching	χ2(1)	*p*
**Chest Pain**				
*proportion*	.61	.89	9.62	002**
*total-n*	92	44	–	–
**Sores**				
*proportion*	.58	.84	7.39	.007**
*total-n*	82	44	–	–
**Tendonitis**				
*proportion*	.69	.91	6.81	.009**
*total-n*	93	44	–	–
**Arthralgia**				
*proportion*	.53	.82	9.94	.002**
*total-n*	91	45	–	–
**Onycholysis**				
*proportion*	.62	.84	5.99	.014^*^
*total-n*	90	45	–	–
**Eczema**				
*proportion*	.61	.84	6.74	.009**
*total-n*	92	45	–	–
**Depression**				
*proportion*	.63	.86	6.52	.011^*^
*total-n*	90	44	–	–
**Migraine**				
*proportion*	.53	.82	10.04	.002**
*total-n*	93	45	–	
**Abdominal Pain**				
*proportion*	.57	.88	11.86	.001**
*total-n*	95	43	–	–

***Note.***

*p <.05;

**p <.01;

***p <.001

### Discussion

Studies 1a and 1b showed that treatment programs that were superior on the quantitative, utility-maximizing attribute of efficacy were more likely to be preferred in a matching task than choice or rating tasks, which did not differ from one another. These findings demonstrate how quantitative, utility-relevant information may dominate preference in matching tasks, while qualitative, affectively-charged attributes may better predict preference in choice and rating tasks. This is consistent with the prior research reviewed above, and extends this finding into the domain of lay medical ethics.

## Study 2: Choice and Rating Versus Matching: Within-Participants

Study 1 demonstrated PRs between choice/rating and matching in a between-participants design. In Study 2, we used a within-subjects design, to address two questions that Study 1 could not: first, do these PRs occur within single individuals, and, second, what trait-level individual differences predict who is most likely to exhibit these PRs? To address these questions, Study 2 employed a multi-session design, in which participants completed a matching task and either a rating or choice task in separate lab sessions. Participants also completed individual difference measures of analytic thinking, thinking style, the Big Five, and moral identity. These measures were respectively selected to investigate a potential link between deliberative, analytical thinking, the self-importance of morality, and personality traits to susceptibility to PRs, and sought to address the question of whether some people are more likely to evince PRs than others. Consistent with the results of Study 1, we predicted that (H1) participants would prefer the higher-efficacy/symptom-present program to the lower-efficacy/symptom-eliminated program significantly more often in the matching task than in the choice and rating tasks, and that (H2a) the choice and rating tasks would not elicit significantly different preferences. We did not formulate any hypotheses regarding potential relationships between individual difference measures and rate of PRs, and included these measures for exploratory purposes. Study 2 was pre-registered at https://aspredicted.org/W6R_DD3. We also included in the pre-registration additional stimuli and predictions from a related project regarding PRs in judgments of sacrificial moral dilemmas, as these stimuli were included in the same lab sessions. Presentation order of the medical stimuli and the sacrificial dilemma stimuli was counterbalanced. Analyses and results for the sacrificial dilemmas are reported elsewhere [52].

### Method

#### Participants.

Participants in Study 2 were recruited from the undergraduate research pool at Nova Southeastern University during the Fall 2022 semester, beginning on September 6^th^, 2022, and ending on December 12^th^, 2022. Participants were awarded course credit upon completion of both sessions of the study. One hundred-sixty-nine participants completed session 1, of which 144 returned to complete session 2, leaving a final sample of *N* = 138 after exclusions (*M*_*Age*_ = 20, *SD*_*Age*_ = 1.11, 112 female).

#### Materials.

The same stimuli from Study 1 were used in Study 2. In addition to the matching and rating or choice tasks, participants completed a six-item Analytic Thinking Scale (ATS) consisting of the three-item Cognitive Reflection Test [105] and three belief-bias syllogisms ([106]; see [107] for evidence that these syllogisms correlate with the CRT; see [52] and [108] for prior use of this six-item scale), the Rational Experiential Inventory (REI; [109]), the Big Five Inventory-2-Short (BFI-2-S; [110]), and the Moral Identity Scale [111]. The ATS was included to test a potential relationship between the tendency to exhibit PRs and the tendency to re-evaluate or reject intuitive responses, while the REI was used to investigate whether the degree to which individuals prefer to rely on intuitive and deliberative thinking strategies predicts PRs. The Moral Identity Scale was intended to investigate a potential relationship between self-importance of moral and ethical principles and the consistency with which participants utilize those principles (i.e., people for whom morality is especially important may be less likely than others to exhibit PRs about moral issues). The BFI2-S was included to explore relationships between broad personality dimensions and PRs.

#### Procedure.

Participants were assigned to two of three tasks. All participants completed the matching task, and either the rating or the choice task. The study was conducted over two sessions separated by at least 13 days. This interval was intended to reduce explicit memory of prior responses and thus reduce consistency pressure. Participants completed one judgment task, two individual difference measures, and a basic demographics survey in the first session, and the other judgment task and the remaining two individual difference measures in the second session. In other words, choice versus rating was manipulated between-subjects, whereas matching versus choice/rating was manipulated within-subjects. Both lab sessions also included other, unrelated tasks. Order of presentation of the different tasks was counterbalanced.

#### Analysis plan.

The proportion of trials for which a participant preferred the higher-efficacy/symptom- present program over the lower-efficacy/symptom-absent program was calculated as in Study 1. This dependent variable was used to test for a significant difference in preference across tasks, and had a possible range of 0–1. Again, as in Study 1, the choice and rating conditions would be combined if they did not significantly differ, and assessed separately if they did.

We next computed a measure capturing the relative frequency of predicted PRs compared to unpredicted, “opposite” reversals. For each participant, trials showing predicted reversals were coded as 1, trials with no reversal (i.e., preference was consistent across choice/rating and matching) were coded as 0, and unpredicted reversals were coded as -1. These coded values were summed; because there were nine program-pairs, this measure had a possible range of -9 (representing unpredicted reversals for all program pairs) to 9 (representing predicted reversals for all program pairs). A positive value on this measure indicates that a participant expressed predicted PRs more often than unpredicted reversals, while a negative value indicates the opposite, and a value of zero indicates that the two types of reversal were expressed equally often. We term this measure “susceptibility to PRs”, consistent with prior work from our lab group [52]. We focus on this coding scheme because it is the most conservative test of our hypothesis. However, we also tested alternative coding schemes in which unpredicted, opposite PRs were coded as 0, which can be found in S3 Text File in the Supporting Information– the results were substantively identical. To test the prevalence of PRs, we used a single-sample *t-*test to assess the mean on the measure of susceptibility to PRs. Finally, the individual difference measures were scored and correlated to the PR susceptibility measure. In order to score the ATS, first, the most common answers were automatically scored using Microsoft Excel (e.g., in the “bat and ball problem”, “5 cents” was scored as correct and “10 cents” was scored as incorrect). Second, answers that were not scoreable automatically were coded by two research assistants. The two research assistants agreed in all but one case; this case was decided by the authors.

### Results

#### Preliminary analyses.

The six items of the ATS showed acceptable internal consistency, α =.68. A composite score for the ATS was produced by summing the number of correct responses, from 0 to 6. The subscales of the REI, Need for Cognition and Faith in Intuition, showed good internal reliability, αs =.82 and.85, respectively. The Internalization subscale of the MIS, which regards the self-importance of moral characteristics, and the Symbolization subscale, which “taps a sensitivity to the moral self as a social object whose actions in the world can convey that one has [moral] characteristics” [111], demonstrated good internal reliability, αs =.85 and.78, respectively. The subscales of the BFI2-S, Extraversion, Conscientiousness, Openness, Agreeableness, and Neuroticism, showed acceptable internal consistency, αs =.75,.72,.63,.69, and.81, respectively. We therefore averaged together the responses on each subscale of these three measures.

Participants’ preferences again did not significantly differ between rating (*M* =.42, *SD* =.34) and choice (*M* =.46, *SD* =.33) tasks between-participants, *t*(136) = 0.64, *p* =.526, *d* = 0.11, supporting H2a. We again followed up on this null-hypothesis test by conducting a Two One-Sided Tests (TOST) equivalence test. This analysis was not pre-registered, but sheds light on whether the two conditions can be considered statistically equivalent to one another. We again conducted a sensitivity analysis to determine the smallest effect size of interest implied by our final sample size. We determined that we had 80% power to detect an effect size of *d* = 0.48, so we set this as the SESOI and set the equivalence bounds in the TOST at ±0.48. The TOST was significant, *t*(136) = 2.18, *p* =.015, so we reject the null hypothesis that there is a true mean difference larger than the SESOI. Once again, we cannot rule out a true effect of small-to-medium size, but we can say with confidence that it is highly unlikely that there is a true effect large enough to be reliably detected in our sample. Also similar to Study 1, the actual observed effect size, *d* = 0.11, is much smaller than the SESOI, is very small in an absolute sense, and would require a prohibitively large sample size of *N* = 2598 to detect with 80% power. Further, while the mean in the rating condition (*M* =.62, *SD* =.27) was nonsignificantly higher than the choice condition (*M* =.57, *SD* =.34) in Study 1, in Study 2 the mean in the rating condition (*M* =.42, *SD* =.34) was nonsignificantly lower than the choice condition (*M* =.46, *SD* =.33), showing there was not a non-significant trend between rating and choice PRs across studies. Looking at the null hypothesis tests, TOSTs, and observed effect sizes, we think it is reasonable to conclude that expressed preferences in the choice and rating condition are meaningfully similar. So, following our preregistered analysis plan, the choice and rating conditions were again combined to form a new choice/rating condition for comparison to the matching condition.

#### Within-subjects analyses.

As predicted (H1), participants preferred the higher-efficacy/symptom-present programs more often in matching (*M* =.59, *SD* =.41) than in choice/rating (*M* =.44, *SD* =.33), *t*(137) = 3.65, *p* <.001, *d*_*RM*_ = 0.31. Note that *d*_*RM*_ denotes the repeated-measures Cohen’s *d,* calculated as the mean difference score of the sample, divided by the standard deviation of difference scores (see [112]).

Table 2 shows the frequency of each possible pattern of judgment for each program pair. For every program pair, the predicted reversal was notably more common than the “opposite” reversal (i.e., preferring the higher-efficacy/symptom-present program in choice/rating, but preferring the lower-efficacy/symptom-eliminated program in matching, see S7 and S8 Tables in the Supporting Information for separate choice and rating condition to matching condition comparisons).

**Table 2:** Proportions of Response Type by Item.

Program Pair	Predicted PRs	Opposite PRs	No PR High-Efficacy Preferred	No PR Low-Efficacy Preferred	Total Responses (Ties Excluded)
Chest Pain	.33	.14	.26	.26	117
Sores	.29	.22	.23	.37	109
Tendonitis	.31	.17	.33	.19	111
Arthralgia	.24	.20	.38	.18	108
Onycholysis	.27	.13	.31	.29	119
Eczema	.31	.18	.28	.23	116
Depression	.27	.14	.29	.30	115
Migraine	.30	.14	.22	.34	111
Abdominal Pain	.25	.20	.34	.21	110

Note: Predicted PRs represent the proportion of participant responses in which the higher-efficacy/symptom-present program is preferred in matching, but the lower-efficacy-symptom/eliminatedprogram is preferred in choice or rating

A single-sample *t-*test with a null-hypothesis mean (μ = 0) confirmed that participants showed a significant tendency to express hypothesized PRs rather than unhypothesized PRs, *t*(137) = 2.99, *p* =.003, *d* = 0.25 (see S3 Text file in the Supporting Information for tests of alternative coding schemes). However, for each item, between 13 and 22 percent of participants demonstrated the opposite, unexpected pattern of PRs. These responses reflect a consistency in responses over nine to eighteen trials of mixed-attribute assessment and may be due to intrapersonal variability in participant attention, intrapersonal variability in the use of judgmental heuristics, such as choosing the option with a greater quantitative value, or intrapersonal variability in participants’ explicit considerations during the study. Nevertheless, the predicted PRs were far more common than the unpredicted PRs, demonstrating a systematic bias in judgment.

We next correlated this measure with the individual difference measures described above. Of these, only the ATS and the Faith in Intuition subscale of the REI significantly predicted susceptibility to PRs: participants with higher ATS scores showed fewer PRs, *r*(136) = −.25, *p* =.002, and participants with higher Faith in Intuition scores showed more PRs, *r*(136) =.18, *p* =.031 (see Table 3 for full correlation matrix; see S9 Table in the Supporting Information for alternative PR coding). As might be expected, Faith in Intuition scores significantly negatively correlated with ATS scores, *r*(136) = −.20, p =.021

**Table 3:** Correlation Matrix, Study 2.

Measure	1	2	3	4	5	6	7	8	9	10	11
1. Susceptibility to Reversals	−	−.01	−.05	−.14	.05	.13	−.03	.18*	.07	.05	-.25**
2. BFI-Openness		−	.25**	.21*	.11	−.04	.41**	.32**	.17*	.14	-.08
3. BFI-Conscientiousness			−	.40**	.36**	−.35**	.36**	.08	.24**	.26**	-.05
4. BFI-Extraversion				−	.09	−.36**	.22**	.19*	.12	.36**	.07
5. BFI-Agreeable					−	−.13	.09	.18*	.32**	.32**	-.03
6. BFI-Neuroticism						−	−.33**	.10	.13	-.08	-.09
7. REI-Rational							−	.06	.11	.09	.07
8. REI-Experiential								−	.29**	.26**	-.20*
9. MIS-Internalization									−	.43**	.08
10. MIS-Symbolization										–	-.05
11. ATS											–

*Note.* The matrix above contains correlations between participant preference reversals, participant scores on the subsections of the Big-Five-Inventory-2S, subsections of the Rational Emotive Inventory, subsections of the Moral Identification Scale, and the Analytic Thinking Scale

*p <.05 (two-tailed),

**p <.01 (two-tailed).

### Discussion

Study 2 demonstrated that the PRs observed in Study 1 can also occur within the same individual. Similar to previous findings, a matching task led participants to rely on utility-related quantitative information (i.e., treatment efficacy), while choice and rating tasks encouraged consideration of qualitative attributes (i.e., patient suffering). We also found that susceptibility to PRs was negatively correlated with analytic thinking, and positively correlated with Faith in Intuition.

## Study 3: Joint and sequential evaluation between-participants

In Study 3, we turn to investigating PRs across evaluation modes rather than elicitation tasks, utilizing a single rating task across either joint or sequential evaluation of stimulus items. We also introduce a new manipulation intended to test if omission bias depends on evaluation mode. Omission bias describes a tendency to express preferences for harm caused by omission of an action over harm caused by an action [113–117]. In a medical context, researchers have shown a tendency for patients (and their guardians, in the case of children) to prefer worse harm caused by an omission of action over less severe harm caused by the commission of an action [117–120], such as the preference to refuse a vaccine that entails minimal risk, but prevents a common and dangerous illness. Omission bias in the context of medical decisions appears to be a robust phenomenon in that it affects judgments of the self [117,121], as well as others [118,119], and appears in both within- and between-participant designs [113,121,122]. However, little prior research [121,122] has examined whether this tendency is affected by differences in evaluation mode. Investigating this question is a secondary aim of Study 3.

Participants in Study 3 rated nine program-pairs, as in Studies 1–2; however, they either viewed the program-pairs jointly (i.e., a pair of programs appeared on each page) or sequentially (i.e., each page presented a single program). Additionally, qualitative attributes (which were presented as symptoms in Studies 1–2) were sometimes described as side-effects actively caused by the treatment. We predicted that when symptoms are the result of omission of action, higher-efficacy/symptom-present programs will be judged more ethical than lower-efficacy/symptom-eliminated programs when rated in joint evaluation, while lower-efficacy/symptom-eliminated programs will be judged more ethical than higher-efficacy/symptom-present programs when rated in in sequential evaluation (H3). We also predicted that: H4) When detracting qualitative outcomes are presented as a side-effect caused by administration of treatment (action) rather than a symptom of an illness resulting from lack of treatment (omission), PRs will be eliminated, and participants will judge the treatment without side-effects as more ethical across both joint and sequential evaluation.

Study 3 was pre-registered at https://aspredicted.org/M6Y_Z77.

### Method

#### Participants.

Two hundred forty-nine adults living in the US were recruited on MTurk on August 4^th^, 2022, and compensated $1 for participation. Due to a miscommunication between members of the research team, data was collected from 34 more participants than had been indicated in the preregistration (https://aspredicted.org/M6Y_Z77). However, only 126 participants successfully completed a comprehension check at the beginning of the questionnaire. Following our pre-registration, we excluded participants who failed this check, resulting in a final sample of *N* = 126. The results are substantively identical when all participants are included in the analyses (see the S10 and S11 Tables in the Supporting Information for full results before and after exclusions). We did not collect demographic information in this study, but our experience with this platform suggests that the sample was likely roughly evenly split between men and women, with a mean age in the mid-thirties.

#### Materials.

**Fig 2:**
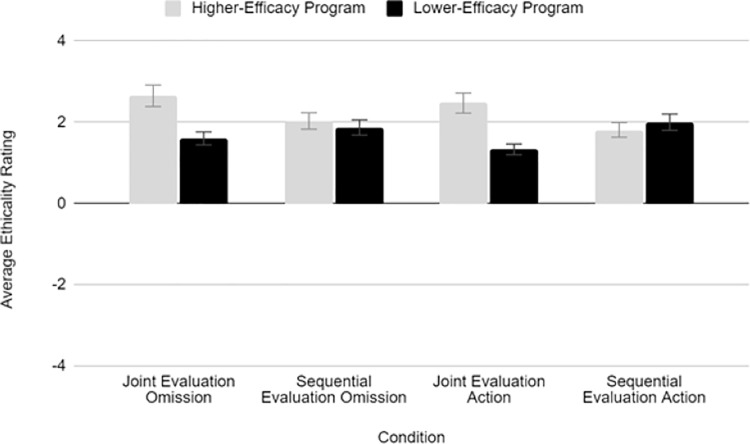
Example of an Item from the Commission Condition.

The same set of nine program-pairs that were used in Studies 1 and 2 were used in Study 3. These nine program-pairs describing the presence or alleviation of a symptom were considered the “harm by omission” stimuli because if patients did not receive the treatment that eliminated a symptom, they would be harmed by experiencing the symptom (i.e., harm is incurred by choosing to forego a treatment that would have prevented it). Nine additional versions of the pairs were developed with the same outcomes as the original treatment programs, but the unpleasant symptoms were described as being the result of an action (i.e., a side effect resulting from implementation of the treatment; see Fig 2 for an example). These were considered the “harm by commission” stimuli because if patients received the treatment, they would be harmed by experiencing the side-effect (i.e., harm is incurred by choosing to implement a treatment).

#### Procedure.

Study 3 study employed a 2 (evaluation mode: sequential versus joint) x 2 (harm: commission versus omission) x 2 (program: higher- versus lower-efficacy) mixed design, with the last factor within-subjects. The instructions, practice items, and exclusions were similar to those in Study 1.

#### Analysis plan.

Unlike in Studies 1 and 2, mean participant ratings for the high and low efficacy programs were utilized as a dependent variable rather than proportion of preference for the higher-efficacy program across trials. The results of Study 3 were analyzed with a mixed ANOVA, with follow-up t-tests to decompose significant interactions.

### Results

We observed a significant main effect of efficacy, *F*(1, 122) = 9.70, *p* =.002, η^2^_p_ =.07, such that the higher-efficacy/ symptom-present programs were rated more ethical overall, but no main effect of evaluation mode, *F*(1, 122) = 0.13, *p* =.723, η^2^_p_ =.001, or harm, *F*(1, 122) = 0.34, *p* =.559, η^2^_p_ =.003.

**Fig 3:**
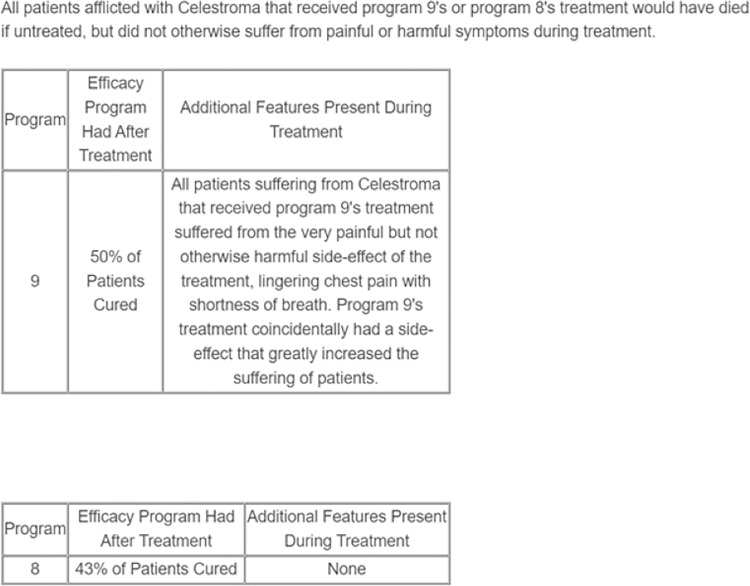
Condition Means, Study 3. **Note:** Error bars represent standard errors.

We also observed a significant evaluation mode x efficacy interaction, *F*(1, 122) = 10.35, *p* =.002, η^2^_p_ =.078), indicating that preferences for one program over the other depended on whether they were evaluated sequentially or jointly. We decomposed this interaction using follow-up *t-*tests. As can be seen in Fig 3, participants did not rate the higher- and lower-efficacy programs significantly differently in sequential evaluation, *t*(76) = 0.136, *p* =.893, *d*_*RM*_ = 0.02, but they preferred the higher-efficacy programs in joint evaluation, *t*(48) = 3.41, *p* <.001, *d*_*RM*_ = 0.49. No other interactions were significant, *p*s >.520. Table 4 presents the results of comparisons between high and low efficacy programs in each condition (see S12 Table in the Supporting Information for item-by-item comparisons between conditions). Overall, participants rated the higher-efficacy program as more ethical in joint evaluation, but were largely indifferent between the two programs in sequential evaluation.

**Table 4:** *t*-tests Between High and Low Efficacy Programs in Both Presentation Mode Conditions Collapsing Across the Action and Omission Manipulation.

Study 3										
Joint-Evaluation					Sequential-Evaluation					
Program *(High or Low* *Efficacy)*	**High**	**Low**	***t(48)***	***p***	***d***_***RM***_	**High**	**Low**	***t(76)***	***p***	***d***_***RM***_
Chest Pain	6.53 (1.83)	5.55 (2.32)	2.07	.044*	0.30	5.75 (2.07)	5.91 (2.12)	−0.57	.571	0.06
Sores	6.41 (1.97)	5.73 (1.93)	1.50	.141	0.21	5.16 (1.86)	6.14 (2.56)	0.04	.283	0.19
Tendonitis	6.86 (1.40)	5.57 (2.20)	3.24	.002**	0.46	6.14 (1.81)	5.99 (2.04)	0.64	.526	0.07
Arthralgia	6.69 (1.43)	5.55 (2.09)	2.84	.007**	0.41	6.14 (1.83)	6.06 (2.17)	0.29	.771	0.03
Onycholysis	6.59 (1.73)	5.61 (2.15)	2.19	.033*	0.31	6.10 (1.80)	5.91 (2.13)	1.06	.291	0.12
Eczema	6.73 (1.52)	5.27 (2.16)	3.79	<.001**	0.54	5.88 (2.03)	5.86 (2.16)	0.10	.920	0.01
Depression	6.84 (1.65)	5.10 (2.25)	4.18	<.001**	0.60	5.82 (2.16)	8.82 (2.01)	0.00	.999	<0.01
Migraine	6.41 (1.71)	5.41 (2.22)	2.34	.024*	0.33	5.60 (2.35)	6.84 (1.88)	−0.82	.416	0.09
Abdominal Pain	6.29 (1.43)	5.35 (2.10)	2.42	.019*	0.35	5.65 (2.10)	5.35 (1.98)	1.56	.124	0.18

### Discussion

Higher-efficacy programs were preferred (i.e., rated as more ethical than lower-efficacy programs) more often in joint evaluation than sequential evaluation, regardless of whether they required patients to endure symptoms or side-effects, consistent with H3. H4, regarding the contingent role of omission bias, was not supported. In fact, we did not observe any omission bias in this study at all (i.e., there was no main effect of harm by omission versus commission, nor did this manipulation interact with any other).

The finding that efficacy better predicted ethicality ratings in joint evaluation, and symptom/side-effect outcomes better predicted ratings in sequential evaluations agrees with the evaluability hypothesis [45]. Our results did not support the hypothesis that omission bias would be attenuated or eliminated in JE. However, this does not establish that omission bias is immune to presentation effects, as no omission bias occurred in the present study. This is also not to say that the present study is evidence against the existence of omission bias. In the present study, participants were only presented with programs that contained side-effects *or* symptoms. Omission bias may be inherently comparative, and emerge for preferences among options that involve omission over commission. In the present study, participants were exposed only to stimuli that involved *either* omission or commission. Future research should test the robustness of omission bias within-participants rather than between-participants, and investigate the potential variability of omission bias across tasks that involve viewing two options at once, such as choice, matching, and joint-rating.

Unsurprisingly, the mean ratings of all programs regardless of evaluation mode indicated that participants thought that it was at least somewhat ethical to implement every program they considered. In other words, participants believed that it is better to administer a treatment – *any* treatment – that cures *some* terminally ill patients, rather than to abandon them to their fate. The key finding in this study is not that people want to save lives – of course they do. It is that how different attributes of a treatment (i.e., patients’ chance of survival, or their suffering during treatment) influence ethicality judgments depends on presentation mode.

## General discussion

The present research demonstrates that elicitation task and evaluation mode have predictable impacts on ethicality judgments in the domain of medical ethics. We thus add to a small but growing literature indicating that moral and medical judgments are susceptible to domain-general preference reversals (PRs).

## Limitations

About a third of participants’ data was excluded from Studies 1a and 1b, and about half of participants’ data was excluded from Study 3 due to failing the comprehension check at the beginning of the questionnaire (See S10 Table in the Supporting Information for analyses including all participants). This may be due to the complexity of the task and information given to participants, though similar tasks have been employed in judgment research with success for decades. The number of exclusions may also be due to the nature of the MTurk samples. While most research indicates that the quality of self-report MTurk data is equivalent to the quality of undergraduate research samples (assuming good data screening procedures) [112,123–130], recent research has raised questions about the validity of data gathered via MTurk [131–134]. However, even critics of MTurk recognize that proper data screening greatly increases the reliability and validity of MTurk data [131,134]. It is also possible that because participants were told the comprehension check was a “warm-up” question, they believed that their response to that item would not “count”, and so responded arbitrarily. Regardless, the results of Study 1 were replicated within-subjects in Study 2, the results of Study 3 are consistent with prior research.

The scope of our studies is limited in two important respects. First, while our participants in Studies 1 and 3 were recruited online from multiple regions, our samples were limited to adults in the United States. As of now, cross-cultural research on PRs across elicitation tasks and evaluation modes is basically nonexistent, so investigating whether the PRs we have observed (and others) occur in other cultural contexts is a crucial direction for future research. If the PRs we have observed in the present research replicate across cultures, this would suggest that moral judgments are widely susceptible to influences of task and presentation mode. Second, our studies only deal with preferences for novel treatments that our participants have never encountered before. Well-rehearsed preferences are more stable than novel judgments [44]; people tend to perseverate judgments they have made many times before. But, when presented with a moral situation without precedent or relevant rehearsed cognitions, individuals are more susceptible to heuristic strategies that produce PRs between presentation modes and elicitation tasks. Thus, our findings mainly apply to situations where one faces novel treatments about which one must form a preference. This includes real-world circumstances such as when new treatments enter the market, forcing patients, practitioners, and policymakers to reevaluate their preferences.

Because H2a, comparing rating and choice, was a null-effect hypothesis, it is possible that there are some differences in preference between rating and choice tasks that we failed to detect due to an insufficiently large sample. In addition to larger samples, future research investigating potential subtle differences between these tasks might not only examine participants’ ultimate judgments, but also probe directly how they are arrived at, via process tracing measures, such as eye-tracking or think-aloud protocols. However, in the present research, at least, we did not find any difference between choice and rating tasks, a result that is largely consistent with prior work [52,66,75–77].

Our stimuli always presented efficacy in a numeric format and quality-of-life information using a qualitative description. They thus confound efficacy with quantitative information and quality-of-life with qualitative information. This is closely related to a common confound in moral psychology research more broadly, in which utilitarian considerations (e.g., lives saved) are confounded with quantitative information and deontological considerations (e.g., directness of harm) are confounded with qualitative information [17,52,135–137]. As in this prior work, we do not think this is especially problematic here, because treatment efficacy is inherently quantitative (i.e., it just *is* the percentage of patients’ lives saved, by definition), while quality of life is more about the patient’s subjective, qualitative experience. However, future research could present quality-of-life information in a quantitative format (e.g., one medical program causes symptoms patients rated as a 5 out of 10 on a pain-and-discomfort scale, while another causes symptoms patients rated as a 1 out of 10 on a pain-and-discomfort scale), eliminating this confound.

Lastly, Study 2, which employed a within-subjects design, included a gap of at least 13 days between sessions to minimize memory effects. We made this decision to reduce consistency pressure, but it leaves open the question of whether our effects would replicate if participants were faced with multiple elicitation tasks (or presentation modes) in rapid succession. We suspect that the results might be weaker in such a design, due to the very consistency pressure we hoped to eliminate, but we think our findings would likely still hold. It is true that, in reality, individuals are at least sometimes faced with multiple tasks side-by-side, or with situations in which they must choose *which* task to employ (e.g., “should I just choose between these, or engage in a quantitative analysis?”). We leave the interesting task of investigating such situations to future work.

## Judgment strategies

In the present research, quantitative, utility-relevant information was more predictive of preference in matching than choice, in matching than rating, and in joint evaluation than sequential evaluation. PRs between elicitation tasks are often explained by the Prominence Hypothesis (though there are other, competing explanations, such as Strategy Compatibility, see [66–68,74,79,80]. PRs between presentation modes, on the other hand, are usually explained by the Evaluability Hypothesis. However, we suggest that there is an important similarity among rating, choice, and sequential evaluation tasks, where judgments are primarily driven by easy-to-assess qualitative attributes, and among matching and joint evaluation tasks, where quantitative information is weighed more heavily. Consistent with our literature review above, we propose a parsimonious explanation of all of our results: people rely on simple, heuristic strategies (e.g., lexicographic ordering) in choice, rating, and sequential evaluation, while they employ more complex, quantitative strategies (e.g., inter-attribute comparison) in matching and joint evaluation [44].

Moreover, our finding that analytic thinking is negatively associated with susceptibility to PRs and Faith in Intuition is positively associated with susceptibility to PRs (Study 2) is similar to the finding that Faith in Intuition is positively correlated with susceptibility to PRs in judgments of sacrificial harms [52]. However, while we replicated the correlation between Faith in Intuition and susceptibility to PRs, this previous work did not observe the correlation between analytic thinking and susceptibility to PRs that we did, despite using the same measure of analytic thinking. The reason for this discrepancy is unclear, but, at a high level, our results are consistent, in that at least some measures of thinking style seem to predict PRs. People who think more carefully and less intuitively seem to have more coherent, consistent moral beliefs, whereas people who rely more on intuition seem to be more prone to inconsistencies in their moral judgments. Though fully explicating the reason for this is beyond the scope of this paper, we speculate that different tasks and presentation modes cue different judgment strategies (i.e., they make different strategies “intuitive”, in the moment), resulting in more intuitive thinkers switching between heuristic strategies in choice, rating, and sequential evaluation and quantitative strategies in matching and joint evaluation, while less intuitive thinkers remain more consistent in their judgment strategies.

### Healthcare implications

Some research suggests that economists, healthcare educators, and medical students are no better than laypeople at resisting non-utilitarian heuristics when choosing between options on behalf of others [97,138]. This raises the troubling possibility that healthcare professionals and patients alike may be susceptible to PRs. This would suggest that patients, providers, administrators, and policymakers could not be trusted to accurately indicate a preference for one treatment over another, because either a “true” preference outside of the biasing effects of elicitation task and evaluation mode would not exist, or there would be no way of knowing which task(s) and evaluation mode(s) elicit “true” preferences, as opposed to biased, “untrue” ones.

The duty to “do no harm” is foundational to medical ethics and is one way of ensuring the well-being of individuals is balanced with the well-being of the population. Do-no-harm reasoning is, however, potentially susceptible to the kinds of task effects we have examined here. While there may be cases in which patient suffering is an important ethical consideration, it is troubling to note that such suffering seems to matter *more* in some evaluation modes or judgment tasks than others. Side effects and symptoms may weigh more heavily than lives saved in some circumstances, but not others.

However, task effects may also help administrators and clinicians increase the evaluability of options or the salience of certain features. For example, if one wanted a doctor or patient to focus on subjective quality-of-life, one might present cancer treatment options sequentially. Conversely, if one wanted to prioritize likelihood of survival over quality-of-life, one might present options jointly. This idea is consistent with our findings, but more research, especially naturalistic studies in ecologically valid healthcare settings, is needed to fully investigate it.

While we have reviewed a growing body of literature on PRs in the medical domain, these previous studies have generally focused on helping patients assess healthcare utility by weighing and balancing treatment options like cost, insurance benefits, distance one must travel to receive treatment, and treatment quality [1,48,50,70,71]. Arguably, maximizing one’s own utility in personal healthcare decisions does not have much to do with morality, while decisions about where clinicians and administrators should focus limited resources does. For example, it is arguably a matter of non-moral (if ill-informed) personal prerogative to overweight or underweight dental coverage in choosing an insurance plan. However, overweighting or underweighting a patient’s chance of survival in treatment choice is certainly a matter of ethics. Thus, we extend this literature from personal medical preferences to lay medical ethics.

### Implications for moral psychology

Beyond demonstrating that lay judgments in the domain of medical ethics are susceptible to PRs, our results raise two broader implications for research in moral psychology. First, they suggest that moral psychologists should be cautious about generalizing from responses to one evaluation mode or elicitation task to draw conclusions about “morality” or “moral judgment” generally [see also 52]. A great deal of research elicits moral judgments using either choice or rating tasks, usually with stimuli presented between-subjects (i.e., in separate evaluation). But, if the judgments that participants render are highly dependent on the design of the study, and would differ in other tasks or presentation modes, then it only makes sense to say that we have learned something about moral judgment *in a specific task and presentation mode,* rather than something about moral judgment generally. Our findings suggest that many past results should probably be reinterpreted in this way.

More broadly, our studies illustrate how methods and theories from outside of moral psychology can be useful in illuminating moral phenomena. Many theoretical approaches to understanding moral psychology are founded on domain-specific theories that posit *unique* cognitive processes in the moral domain. We have drawn methods and theoretical insights from outside of moral psychology to show that moral preferences depend importantly on how they are elicited, just as other kinds of preferences do. We think that future research in moral psychology would benefit from drawing more frequently on research from other areas such as consumer behavior research, rather than relying solely on theories and methods that are narrowly restricted to morality.

## Conclusion

Like the study of economics, the study of medical judgment and decision making has generally assumed that ethical judgments are consistent across variations in the presentation of identical stimuli or across different ways of measuring them. The present study offers evidence to the contrary by demonstrating classic preference reversals in ethicality judgments of medical treatments. In agreement with two well-known explanations for PRs, the evaluability effect and the Prominence Hypothesis, the present findings also highlight the apparent salience of evaluable quantitative information in matching and joint evaluation tasks, and the contrasting salience of qualitative information in choice, rating, and separate evaluation tasks.

In their book reviewing research on the fragility and formation of preferences, Lichtenstein and Slovic [40] suggest that because preferences have been found to be dependent on elicitation and evaluation modes, they are in fact constructed on-the-fly rather than being established, stable opinions. We see no reason to think that most ethical preferences are determined exclusively by any special set of heuristics or principles that apply only to moral thinking. Thus, the present research provides support for the idea that at least some moral judgments are constructed on-the-fly relative to task and presentation of stimuli, just as other sorts of preferences are.

## Supporting information

S1 Text Pretests and Final Stimuli. (PDF)

S2 Text Study 1a and 1b Preregistered Analyses. (PDF)

S3 Text Study 2, Alternative Reversal Coding. (PDF)

S1 Fig Instructions. (PDF)

S2 Fig Practice/Comprehension Check. (PDF)

S3 Fig Stimuli Symptom Pair 1. (PDF)

S4 Fig Stimuli Symptom Pair 2. (PDF)

S5 Fig Stimuli Symptom Pair 3. (PDF)

S6 Fig Stimuli Symptom Pair 4. (PDF)

S7 Fig Stimuli Symptom Pair 5. (PDF)

S8 Fig Stimuli Symptom Pair 6. (PDF)

S9 Fig Stimuli Symptom Pair 7. (PDF)

S10 Fig Stimuli Symptom Pair 8. (PDF)

S11 Fig Stimuli Symptom Pair 9. (PDF)

S12 Fig Stimuli: Symptom Pair 9, Matching: Low-Efficacy Condition. (PDF)

S13 Fig Stimuli: Symptom Pair 9, Matching: High-Efficacy Condition. (PDF)

S14 Fig Stimuli: Symptom Pair 9, Rating/Joint Evaluation. (PDF)

S15 Fig Stimuli: Symptom Pair 9, Sequential Evaluation, High-Efficacy Item. (PDF)

S16 Fig Symptom Pair 9a, Sequential Evaluation High-Efficacy/Side-Effect Item. (PDF)

S1 Table Pretest 1: Sequential Rating Symptoms Means. (PDF)

S2 Table Pretest 2: Joint Rating Efficacy Means. (PDF)

S3 Table Study 1a and 1b Separate and Combined Analyses by Item After Exclusions. (PDF)

S4 Table Proportion of Participants Indicating the Higher-efficacy/symptom-present Program is More Ethical in Study 1 Before Exclusions. (PDF)

S5 Table Proportion of Participants Indicating the Higher-efficacy/symptom-present Program is More Ethical in Study 1 in Choice vs Matching. (PDF)

S6 Table Proportion of Participants Indicating the Higher-efficacy/symptom-present Program is More Ethical in Study 1 in Rating vs Matching. Study 2 Supplemental Analyses.
(PDF)

S7 Table Proportion of Response Type in Study 2 by Item in the Choice and Matching Conditions. (PDF)

S8 Table Proportion of Response Type in Study 2 by Item in the Rating and Matching Conditions. (PDF)

S9 Table Correlation Matrix, Study 2, PR Susceptibility Coding Scheme 2. Study 3 Supplemental Results.
(PDF)

S10 Table Effects of Presentation Mode, Efficacy, and Act/Omission Manipulations on Ratings Before Exclusions. (PDF)

S11 Table Effects of Presentation Mode, Efficacy, and Act/Omission Manipulations on Ratings After Exclusions. (PDF)

S12 Table Independent Samples t-tests by Program Across Condition Collapsing Across the Action and Omission Manipulation. (PDF)
