# Supplementary material for: Preference reversals in ethicality judgments of medical treatments
Source: PLoS One. 2025 Apr 29;20(4):e0319233. doi: 10.1371/journal.pone.0319233 (PMC12040148; doi:10.1371/journal.pone.0319233)
Supplement: S1 Text — (PDF) [file pone.0319233.s001.pdf]

Text S1. Pretests and Stimuli.

Pretest 1. Participants (*N* = 63) were recruited from MTurk and compensated $1 for completing the first pretest. The first pretest examined mean ethicality ratings for the higher-efficacy/symptom-present item, and lower-efficacy/symptom-eliminated item for twenty-one pairs of medical programs in sequential evaluation. Each pair of programs was intended to measure the impact of the described symptom on the rating of the programs. Low efficacy programs pseudo-randomly ranged from 49% effective to 52% effective, such that each efficacy was presented an equal number of times. High efficacy programs ranged from 57% effective to 60% effective. Efficacy was varied to avoid habitual responses to nearly identical items. Small differences in efficacies were chosen to avoid overwhelming the effect of symptom, and because real medical treatments often pose tradeoffs between undesirable outcomes. In order to keep the questionnaire a reasonable length, participants were administered a subset of programs, and received either ten or eleven program-pairs, each describing one symptom.

Before evaluating the focal stimuli, participants were given an example program with very high efficacy that alleviated symptoms, and an example program with very low efficacy that presented symptoms, with suggested ratings of +4 and -4 respectively to help participants familiarize themselves with the response scale. Participants were then given a "practice round" where they again evaluated programs with highly desirable and highly undesirable outcomes (see Supplement for materials). If a participant expressed a preference for the program with inferior outcomes in the practice rounds, data from that participant was excluded, because they apparently were not paying attention and/or did not understand the task.

Results showed that for nine of the twenty-one symptoms tested, the lower-efficacy/symptom-eliminated program was rated as more ethical than the higher-

efficacy/symptom-present program, on average. These symptoms (tendinitis, ocular migraine, chronic depression, lingering chest pain and shortness of breath, eczema, onycholysis, abdominal pain, arthralgia, and painful sores in and around the mouth) were selected for use in the second pretest and the final stimuli. Means for all symptoms are presented in Table S1.

Pretest 2. Participants (*N* = 62) were recruited from MTurk and compensated $0.50 for participation in the second pretest. The second pretest assessed the strength of the efficacy manipulation on ratings in joint evaluation of both programs in each pair. The goal of the second pretest was to assess the strength of the efficacy attribute in our stimuli. Huber et al. [1] found that equivalent differences on quantitative attributes in a decision-making task between multi-attribute options for ski vacations mattered more when they were on the "low end" (i.e. an undesirable value) of possible values than the "high end" (i.e. a desirable value). For example, a difference in chance of good snow between 11% and 13% would be perceived as larger or more important than the difference between 87% and 89%. If general principles of judgment apply to moral judgments specifically, then it might be expected that the same pattern would emerge in the present research. Thus, by random assignment, half of the participants were presented with pairs of low-efficacy (40% to 52%) programs with 5% maximum possible difference in efficacy within each program type (high or low efficacy), and a possible difference of 5%-11% between programs in each pair. The other half of the participants were presented with pairs of high-efficacy (78% to 90%) programs with the same symptoms and the same ranges of efficacy within program-types and between program-types. In the low-efficacy group, the higher-efficacy/symptom-present program was preferred over the lower-efficacy/symptom-absent program for every symptom. In the high-efficacy group, the higher-efficacy/symptom-present program was preferred for only five out of nine pairs. In line with prior research, the "lower"

efficacy range was used in the final set of stimuli, as it constituted a psychologically stronger manipulation. Means for all program-pairs are presented in Table S2.

Final Stimuli. Final stimuli were constructed from the symptoms tested in Table S1, and the efficacy ranges tested in Table S2, as described in the pretests. Figures S1-S16 are images of the final stimuli as participants viewed them.
