# Supplementary material for: Preference reversals in ethicality judgments of medical treatments
Source: PLoS One. 2025 Apr 29;20(4):e0319233. doi: 10.1371/journal.pone.0319233 (PMC12040148; doi:10.1371/journal.pone.0319233)
Supplement: S2 Text — (PDF) [file pone.0319233.s002.pdf]

Text S2. Study 1 Supplemental Analyses.

Study 1a and 1b Preregistered Analyses

Consistent with H2, participants' rate of preference for the higher-efficacy/symptom-present program over the low-efficacy/symptom-absent program did not differ significantly between the choice and rating conditions in Study 1a, $M1 = .57$, $SD1 = .35$, $M2 = .59$, $SD2 = .29$, $t(62) = 0.35$, $p = .729$, $d = 0.09$, or Study 1b, $M1 = .58$, $SD1 = .34$, $M2 = .66$, SD 2= .24, $t(36) = 0.86$, $p = .393$, $d = 0.28$, so the choice and rating conditions were collapsed to form a combined choice/rating condition for each study. Consistent with H1, participants preferred the higher-efficacy/symptom-present program more often in the matching condition ($M = .79$, $SD = .38$) than in the combined choice/rating condition ($M = .58$, $SD = .32$) in Study 1a, $t(82) = 2.43$, $p = .017$, $d = 0.53$. Similarly, participants preferred the higher-efficacy/symptom-present program more often in the matching condition ($M = .90$, $SD = .27$) than in the combined choice/rating condition ($M = .63$, $SD = .29$) in Study 1b, $t(61) = 3.82$, $p < .001$, $d = 0.96$.
