# Supplementary material for: Preference reversals in ethicality judgments of medical treatments
Source: PLoS One. 2025 Apr 29;20(4):e0319233. doi: 10.1371/journal.pone.0319233 (PMC12040148; doi:10.1371/journal.pone.0319233)
Supplement: S3 Text — (PDF) [file pone.0319233.s003.pdf]

Text S3. Study 2 Supplemental Analyses.

Regardless of whether a coding scheme respectively assigned the values of –1, 1, or 0, $t(137) = 2.99$, $p = .003$, $d = 0.25$, 1, 1, or 0, $t(137) = 15.71$, $p < .001$, $d = 1.34$, or 0, 1, or 0, $t(137) = 9.65$, $p < .001$, $d = 0.82$, to code the each trial in which a participant expressed an unpredicted PR, a predicted PR, and no PR, a t-test against the null mean of 0 was significant.
