# Supplementary material for: Preference reversals in ethicality judgments of medical treatments
Source: PLoS One. 2025 Apr 29;20(4):e0319233. doi: 10.1371/journal.pone.0319233 (PMC12040148; doi:10.1371/journal.pone.0319233)
Supplement: S1 Fig — (PDF) [file pone.0319233.s004.pdf]

Figure S1

Instructions

Please read the instructions below:

Healthcare professionals are often presented with difficult decisions about what medical programs to implement. On the next several pages, you will be presented with treatment plans that were implemented at similar but different hospitals to help patients suffering from advanced Celestroma, a rare cancer in its final stages. You will be presented with information about treatment programs, two at a time, and asked to pick which program was more ethical for medical professionals to choose for funding. Consider that in these scenarios all patients that were not treated died. Though this survey does not present real information about actual treatment programs, dilemmas like this are faced by human beings every day when clinicians and policy makers must choose between projects to fund. Please click the arrow button to continue.

→
