# Supplementary material for: Preference reversals in ethicality judgments of medical treatments
Source: PLoS One. 2025 Apr 29;20(4):e0319233. doi: 10.1371/journal.pone.0319233 (PMC12040148; doi:10.1371/journal.pone.0319233)
Supplement: S2 Fig — (PDF) [file pone.0319233.s005.pdf]

Figure S2

Practice/Comprehension Check item for Studies 1a and 1b in Choice Condition (Counterbalance Order 1)

Now, try a warm up question. This question will help you familiarize yourself with the rating task before going on to the next question. Note that there is always a sentence above the tables representing the treatment programs that provides further information about the patients in question. Before answering, also note that program 2 has a higher efficacy, and a better symptom outcome than program 3.

All patients afflicted with Celestroma that received Program 2's or Program 3's treatment suffered from the very painful but not otherwise harmful symptom of the disease, frequent headaches.

Program	Efficacy Program Had After Treatment	Additional Features Present During Treatment
2	78% of Patients Cured	Program 2's treatment coincidentally had powerful analgesic (headache relieving) qualities that completely eliminated that symptom, and greatly reduced the suffering of patients.

Program	Efficacy Program Had After Treatment	Additional Features Present During Treatment
3	40% of Patients Cured	None

Which program was more ethical for medical professionals to choose to fund and implement?

Program 2	Program 3
○	○
