# Supplementary material for: Preference reversals in ethicality judgments of medical treatments
Source: PLoS One. 2025 Apr 29;20(4):e0319233. doi: 10.1371/journal.pone.0319233 (PMC12040148; doi:10.1371/journal.pone.0319233)
Supplement: S3 Fig — (PDF) [file pone.0319233.s006.pdf]

Figure S3

Stimuli Symptom Pair 1, Counterbalance Order 1

All patients afflicted with Celestroma that received Program 9's or Program 8's treatment suffered from the very painful but not otherwise harmful symptom of the disease, lingering chest pain with shortness of breath.

Program	Efficacy Program Had After Treatment	Additional Features Present During Treatment
9	50% of Patients Cured	None

Program	Efficacy Program Had After Treatment	Additional Features Present During Treatment
8	43% of Patients Cured	Program 8's treatment coincidentally had powerful muscle-relaxant qualities that completely alleviated patients' chest pain and shortness of breath, and greatly reduced the suffering of patients.
