# Supplementary material for: Preference reversals in ethicality judgments of medical treatments
Source: PLoS One. 2025 Apr 29;20(4):e0319233. doi: 10.1371/journal.pone.0319233 (PMC12040148; doi:10.1371/journal.pone.0319233)
Supplement: S4 Fig — (PDF) [file pone.0319233.s007.pdf]

Figure S4

Stimuli: Symptom Pair 2, Counterbalance Order 1

All patients afflicted with Celestroma that received Program 35's or Program 34's treatment suffered from the very painful but not otherwise harmful symptom of the disease, painful sores in and around the mouth.

Program	Efficacy Program Had After Treatment	Additional Features Present During Treatment
35	52% of Patients Cured	None

Program	Efficacy Program Had After Treatment	Additional Features Present During Treatment
34	48% of Patients Cured	Program 34's treatment coincidentally had powerful qualities that completely alleviated painful sores, and greatly reduced the suffering of patients.
