# Supplementary material for: Preference reversals in ethicality judgments of medical treatments
Source: PLoS One. 2025 Apr 29;20(4):e0319233. doi: 10.1371/journal.pone.0319233 (PMC12040148; doi:10.1371/journal.pone.0319233)
Supplement: S5 Fig — (PDF) [file pone.0319233.s008.pdf]

Figure S5

Stimuli: Symptom Pair 3, Counterbalance Order 1

All patients afflicted with Celestroma that received Program 33's or program 32's treatment suffered from the very painful but not otherwise harmful symptom of the disease, tendonitis.

Program	Efficacy Program Had After Treatment	Additional Features Present During Treatment
33	52% of Patients Cured	None

Program	Efficacy Program Had After Treatment	Additional Features Present During Treatment
32	44% of Patients Cured	Program 32's treatment coincidentally had powerful anti-inflammatory qualities that completely alleviated patients' tendonitis, and greatly reduced the suffering of patients.
