# Supplementary material for: Preference reversals in ethicality judgments of medical treatments
Source: PLoS One. 2025 Apr 29;20(4):e0319233. doi: 10.1371/journal.pone.0319233 (PMC12040148; doi:10.1371/journal.pone.0319233)
Supplement: S6 Fig — (PDF) [file pone.0319233.s009.pdf]

Figure S6

Stimuli: Symptom Pair 4, Counterbalance Order 1

All patients afflicted with Celestroma that received Program 43's or Program 42's treatment suffered from the very painful but not otherwise harmful symptom of the disease, arthralgia (joint pain).

Program	Efficacy Program Had After Treatment	Additional Features Present During Treatment
43	50% of Patients Cured	None

Program	Efficacy Program Had After Treatment	Additional Features Present During Treatment
42	43% of Patients Cured	Program 42's treatment coincidentally had powerful pain-relieving qualities that completely alleviated patients' joint pain, and greatly reduced the suffering of patients.
