# Supplementary material for: Preference reversals in ethicality judgments of medical treatments
Source: PLoS One. 2025 Apr 29;20(4):e0319233. doi: 10.1371/journal.pone.0319233 (PMC12040148; doi:10.1371/journal.pone.0319233)
Supplement: S7 Fig — (PDF) [file pone.0319233.s010.pdf]

Figure S7

Stimuli: Symptom Pair 5, Counterbalance Order 1

All patients afflicted with Celestroma that received Program 39's or Program 38's treatment suffered from the very painful but not otherwise harmful symptom of the disease, onycholysis (painful loss of fingernails and toenails).

Program	Efficacy Program Had After Treatment	Additional Features Present During Treatment
39	51% of Patients Cured	None

Program	Efficacy Program Had After Treatment	Additional Features Present During Treatment
38	44% of Patients Cured	Program 38's treatment coincidentally had powerful nail-protein-fortifying qualities that completely alleviated patients' onycholysis, and greatly reduced the suffering of patients.
