# Supplementary material for: Preference reversals in ethicality judgments of medical treatments
Source: PLoS One. 2025 Apr 29;20(4):e0319233. doi: 10.1371/journal.pone.0319233 (PMC12040148; doi:10.1371/journal.pone.0319233)
Supplement: S8 Fig — (PDF) [file pone.0319233.s011.pdf]

Figure S8

Stimuli: Symptom Pair 6, Counterbalance Order 1

All patients afflicted with Celestroma that received Program 29's or program 28's treatment suffered from the very painful but not otherwise harmful symptom of the disease, painful temporary eczema and temporary skin lesions

Program	Efficacy Program Had After Treatment	Additional Features Present During Treatment
29	49% of Patients Cured	None

Program	Efficacy Program Had After Treatment	Additional Features Present During Treatment
28	41% of Patients Cured	Program 28's treatment coincidentally had powerful dermatological qualities that completely alleviated patients' eczema and skin lesions, and greatly reduced the suffering of patients.
