# Supplementary material for: Preference reversals in ethicality judgments of medical treatments
Source: PLoS One. 2025 Apr 29;20(4):e0319233. doi: 10.1371/journal.pone.0319233 (PMC12040148; doi:10.1371/journal.pone.0319233)
Supplement: S9 Fig — (PDF) [file pone.0319233.s012.pdf]

**Figure S9**

*Stimuli: Symptom Pair 7, Counterbalance Order 1*

All patients afflicted with Celestroma that received Program 11's or Program 10's treatment suffered from the very painful but not otherwise harmful symptom of the disease, chronic depression.

| Program | Efficacy Program Had After Treatment | Additional Features Present During Treatment |
|---|---|---|
| 11 | 50% of Patients Cured | None |

---

| Program | Efficacy Program Had After Treatment | Additional Features Present During Treatment |
|---|---|---|
| 10 | 43% of Patients Cured | Program 10's treatment coincidentally had powerful anti-depressant qualities that completely alleviated patients' depression, and greatly reduced the suffering of patients. |

---
