# Supplementary material for: Preference reversals in ethicality judgments of medical treatments
Source: PLoS One. 2025 Apr 29;20(4):e0319233. doi: 10.1371/journal.pone.0319233 (PMC12040148; doi:10.1371/journal.pone.0319233)
Supplement: S10 Fig — (PDF) [file pone.0319233.s013.pdf]

Figure S10

Stimuli: Symptom Pair 8, Counterbalance Order 1

All patients afflicted with Celestroma that received Program 19's or Program 18's treatment suffered from the very painful but not otherwise harmful symptom of the disease, ocular migraines.

Program	Efficacy Program Had After Treatment	Additional Features Present During Treatment
19	48% of Patients Cured	None

Program	Efficacy Program Had After Treatment	Additional Features Present During Treatment
18	42% of Patients Cured	Program 18's treatment coincidentally had powerful anti-migraine qualities that completely alleviated patients' ocular migraines, and greatly reduced the suffering of patients.
