# Supplementary material for: Preference reversals in ethicality judgments of medical treatments
Source: PLoS One. 2025 Apr 29;20(4):e0319233. doi: 10.1371/journal.pone.0319233 (PMC12040148; doi:10.1371/journal.pone.0319233)
Supplement: S13 Fig — (PDF) [file pone.0319233.s016.pdf]

Figure S13

Stimuli: Symptom Pair 9, Matching: High-Efficacy Condition, Counterbalance Order 1

All patients afflicted with Celestroma that received Program 22's or Program 23's treatment suffered from the very painful but not otherwise harmful symptom of the disease, sharp abdominal pain.

Program	Efficacy Program Had After Treatment	Additional Features Present During Treatment
22	40% of Patients Cured	Program 22's treatment coincidentally had powerful pain-relieving qualities that completely alleviated patients' abdominal pain, and greatly reduced the suffering of patients.

Program	Efficacy Program Had After Treatment	Additional Features Present During Treatment
23	___% of Patients Cured	None

What percent efficacy would program 23 have had to have had to make it equally as ethical as program 22 for medical professionals to choose to fund and implement?
