# Supplementary material for: Preference reversals in ethicality judgments of medical treatments
Source: PLoS One. 2025 Apr 29;20(4):e0319233. doi: 10.1371/journal.pone.0319233 (PMC12040148; doi:10.1371/journal.pone.0319233)
Supplement: S14 Fig — (PDF) [file pone.0319233.s017.pdf]

Figure S14

Stimuli: Symptom Pair 9, Rating/Joint Evaluation, Counterbalance Order 1

All patients afflicted with Celestroma that received Program 23's or Program 22's treatment suffered from the very painful but not otherwise harmful symptom of the disease, sharp abdominal pain.

Program	Efficacy Program Had After Treatment	Additional Features Present During Treatment
23	49% of Patients Cured	None

Program	Efficacy Program Had After Treatment	Additional Features Present During Treatment
22	40% of Patients Cured	Program 22's treatment coincidentally had powerful pain-relieving qualities that completely alleviated patients' abdominal pain, and greatly reduced the suffering of patients.
