# Supplementary material for: Preference reversals in ethicality judgments of medical treatments
Source: PLoS One. 2025 Apr 29;20(4):e0319233. doi: 10.1371/journal.pone.0319233 (PMC12040148; doi:10.1371/journal.pone.0319233)
Supplement: S15 Fig — (PDF) [file pone.0319233.s018.pdf]

Figure S15

Stimuli: Symptom Pair 9, Sequential Evaluation, High-Efficacy/Symptom Item

All patients afflicted with Celestroma that received Program 23's treatment suffered from the very painful but not otherwise harmful symptoms of the disease, sharp abdominal pain.

Program	Efficacy Program Had After Treatment	Additional Features Present During Treatment
23	49% of Patients Cured	None

How ethical was it for medical professionals to choose Program 23 to fund and implement?

Very Unethical -4	-3	Somewhat Unethical -2	-1	Neither Ethical Nor Unethical 0	1	Somewhat Ethical 2	3	Very Ethical 4
