# Supplementary material for: Preference reversals in ethicality judgments of medical treatments
Source: PLoS One. 2025 Apr 29;20(4):e0319233. doi: 10.1371/journal.pone.0319233 (PMC12040148; doi:10.1371/journal.pone.0319233)
Supplement: S16 Fig — (PDF) [file pone.0319233.s019.pdf]

Figure S16

Stimuli: Symptom Pair 9a, Sequential Evaluation High Efficacy/Side-Effect Item

All patients afflicted with Celestroma that received program 23's treatment would have died if untreated, but did not otherwise suffer from painful or harmful symptoms during treatment.

Program	Efficacy Program Had After Treatment	Additional Features Present During Treatment
23	49% of Patients Cured	All patients suffering from Celestroma that received program 23's treatment suffered from the very painful but not otherwise harmful side-effect of the treatment, sharp abdominal pain. Program 23's treatment coincidentally had a side-effect that greatly increased the suffering of patients.

How ethical was it for medical professionals to choose Program 23 to fund and implement?

Very Unethical -4	-3	Somewhat Unethical -2	-1	Neither Ethical Nor Unethical 0	1	Somewhat Ethical 2	3	Very Ethical 4
