# Supplementary material for: Preference reversals in ethicality judgments of medical treatments
Source: PLoS One. 2025 Apr 29;20(4):e0319233. doi: 10.1371/journal.pone.0319233 (PMC12040148; doi:10.1371/journal.pone.0319233)
Supplement: S1 Table — (PDF) [file pone.0319233.s020.pdf]

Table S1. Pretest 1: Sequential Rating Symptoms Means

Symptom	High Efficacy (Symptom Present) Program Mean	Low Efficacy (Alleviated Symptom) Program Mean
Painful Swollen Legs and Ankles	6.80	6.63
Arthralgia	6.43	6.57
Back and Side-Pain	6.50	6.40
Onycholysis	6.43	6.67
Painful Burning Sensation in Legs and Arms	7.03	6.13
Painful Sores In and Around the Mouth	6.50	6.57
Tendinitis	6.00	6.70
Frequent Painful Urination	6.73	6.30
Painful Temporary Eczema	6.60	6.90
Severe Jaw Pain	6.43	6.33
Sharp Abdominal Pain	6.42	6.58
Persistent Heartburn, Resulting from Painful Lesions in the Lower Esophagus	6.81	6.39
Severe Nausea	7.26	6.90
Spasticity	6.35	6.00
Lingering Chest Pain with Shortness of Breath	6.55	6.87
Chronic Depression	6.19	6.52
Paresthesia	6.68	6.52

Stomach Pain, Irritable Bowel Syndrome, and Frequent Bloody Diarrhea.	6.74	6.39
Ocular Migraines	6.29	6.71
Formation of Ulcers on the Vocal Chords, Causing a Sharp Pain While Swallowing, Eating, and Speaking	6.45	6.19
High Fever, Chills, and Heavy Perspiration	6.39	5.94

Note: This table presents means from the first pretest, where symptom manipulations were tested.
