# Supplementary material for: Preference reversals in ethicality judgments of medical treatments
Source: PLoS One. 2025 Apr 29;20(4):e0319233. doi: 10.1371/journal.pone.0319233 (PMC12040148; doi:10.1371/journal.pone.0319233)
Supplement: S2 Table — (PDF) [file pone.0319233.s021.pdf]

Table S2

Pretest 2: Joint Rating Efficacy Means

Symptom	High Efficacy Range		Low Efficacy Range	
	**High Efficacy (Symptom Present) Program Mean**	**Low Efficacy (Alleviated Symptom) Program Mean**	**High Efficacy (Symptom Present) Program Mean**	**Low Efficacy (Alleviated Symptom) Program Mean**
Tendinitis	6.41	6.21	6.88	6.00
Ocular Migraine	6.91	6.50	6.65	5.77
Chronic Depression	6.41	6.24	6.85	5.38
Lingering Chest Pain and Shortness of Breath	6.65	5.88	6.85	5.19
Painful Temporary Eczema	6.65	6.47	6.69	5.96
Onycholysis	6.47	6.44	6.58	5.96
Sharp Abdominal Pain	6.74	6.03	6.78	5.62
Arthralgia	7.18	5.53	7.04	5.85
Painful Sores In and Around the Mouth	6.64	6.41	6.58	6.46

Note: This table presents means from the second pretest, where high ranges for all programs and low ranges for all programs were tested.
