# Supplementary material for: Preference reversals in ethicality judgments of medical treatments
Source: PLoS One. 2025 Apr 29;20(4):e0319233. doi: 10.1371/journal.pone.0319233 (PMC12040148; doi:10.1371/journal.pone.0319233)
Supplement: S3 Table — (PDF) [file pone.0319233.s022.pdf]

Table S3. Proportion of Participants Indicating the Higher-efficacy/symptom-present Program is More Ethical in Study 1 After Exclusions

		**Study 1a**		
Program Pair	Choice/Rating	Matching	χ2(1)	*p*
Chest Pain	.62	.92	5.44	.019*
Sores	.67	.88	2.23	.135
Tendonitis	.68	.96	4.96	.026*
Arthralgia	.56	.88	5.58	.018*
Onycholysis	.61	.88	3.76	.052*
Eczema	.59	.92	6.43	.011*
Depression	.72	.96	3.86	.049*
Migraine	.65	.88	2.99	.084
Abdominal Pain	.59	.92	6.04	.014*
		**Study 1b**		
Program Pair	Choice/Rating	Matching	χ2(1)	*p*
Chest Pain	.60	.84	2.66	.103
Sores	.55	.79	2.78	.108
Tendonitis	.69	.85	1.21	.271
Arthralgia	.51	.75	2.60	.107
Onycholysis	.63	.80	1.32	.252
Eczema	.62	.75	0.60	.438
Depression	.59	.75	1.08	.299
Migraine	.46	.75	4.02	.045*
Abdominal Pain	.56	.84	3.87	.049*

Combined Analyses				
Program Pair	Choice/Rating	Matching	$\chi2(1)$	*p*
Chest Pain	.61	.89	9.62	.002**
Sores	.58	.84	7.39	.007**
Tendonitis	.69	.91	6.81	.009**
Arthralgia	.53	.82	9.94	.002**
Onycholysis	.62	.84	5.99	.014*
Eczema	.61	.84	6.74	.009**
Depression	.63	.86	6.52	.011*
Migraine	.53	.82	10.04	.002**
Abdominal Pain	.57	.88	11.86	.001

Note: Chi-square tests for independence comparing the rating/choice and matching dependent variables for each item failed to reach significance in Study 1b, but all were directionally consistent with H1.
