# Supplementary material for: Preference reversals in ethicality judgments of medical treatments
Source: PLoS One. 2025 Apr 29;20(4):e0319233. doi: 10.1371/journal.pone.0319233 (PMC12040148; doi:10.1371/journal.pone.0319233)
Supplement: S4 Table — (PDF) [file pone.0319233.s023.pdf]

Table S4

Proportion of Participants Indicating the Higher-efficacy/symptom-present Program is More Ethical in Study 1 Before Exclusions

**Study 1a**				
Program Pair	Choice/Rating	Matching	χ2(1)	*p*
Chest Pain	.59	.63	0.04	.842
Sores	.64	63	<0.01	.999
Tendonitis	.66	.70	0.04	.835
Arthralgia	.46	.66	3.25	.072
Onycholysis	.60	.63	0.01	.918
Eczema	.48	.69	3.50	.061
Depression	.65	.68	0.01	.941
Migraine	.59	.63	0.04	.084
Abdominal Pain	.57	.56	0.35	.556
**Study 1b**				
Program Pair	Choice/Rating	Matching	χ2(1)	*p*
Chest Pain	.58	.52	0.18	.671
Sores	.53	.53	0	.999
Tendonitis	.61	.60	<0.01	.999
Arthralgia	.48	.60	1.15	.284
Onycholysis	.57	.57	<0.01	.999
Eczema	.60	.51	0.76	.382
Depression	.61	.60	<0.01	.968
Migraine	.48	.53	0.17	.678
Abdominal Pain	.53	.58	0.17	.679

Combined Analyses

Program Pair	Choice/Rating	Matching	χ2(1)	*p*
Chest Pain	.58	.58	<0.01	.999
Sores	.57	.59	0.02	.881
Tendonitis	.63	.65	0.09	.771
Arthralgia	.47	.63	4.85	.028*
Onycholysis	.58	.60	0.03	.872
Eczema	.56	.60	0.22	.641
Depression	.63	.64	<0.01	.958
Migraine	.51	.58	0.76	.385
Abdominal Pain	.54	.61	0.95	.331

Note: Results from Studies 1a and 1b were nonsignificant before exclusions. A t-test comparing the combined choice and rating conditions to the matching condition failed to reach significance in Study 1a before exclusions, $t(146) = 0.04$, $p = .969$, $d < 0.01$, Study 1b before exclusions, $t(99) = -0.95$, $p = .342$, $d = -0.19$, and the combined data before exclusions, $t(247) = -0.84$, $p = .402$, $d = -0.11$.
