# Supplementary material for: Preference reversals in ethicality judgments of medical treatments
Source: PLoS One. 2025 Apr 29;20(4):e0319233. doi: 10.1371/journal.pone.0319233 (PMC12040148; doi:10.1371/journal.pone.0319233)
Supplement: S5 Table — (PDF) [file pone.0319233.s024.pdf]

Table S5. *Proportion of Participants Indicating the Higher-efficacy/symptom-present Program is More Ethical in Study 1 in Choice vs Matching*

**Study 1a**				
Program Pair	Choice	Matching	$\chi2(1)$	*p*
Chest Pain	.58	.84	2.60	.020*
Sores	.52	.79	2.67	.103
Tendonitis	.71	.85	0.67	.415
Arthralgia	.48	.75	2.54	.111
Onycholysis	.65	.80	0.76	.384
Eczema	.61	.75	0.50	.478
Depression	.55	.75	1.34	.247
Migraine	.48	.75	2.54	.111
Abdominal Pain	.52	.84	4.11	.043*
**Study 1b**				
Program Pair	Choice	Matching	$\chi2(1)$	*p*
Chest Pain	.65	.92	3.28	.070
Sores	.47	.88	6.43	.011*
Tendonitis	.59	.96	6.48	.011*
Arthralgia	.65	.88	2.02	.155
Onycholysis	.59	.88	3.28	.070
Eczema	.47	.92	8.38	.004*
Depression	.71	.96	3.26	.071
Migraine	.47	.88	6.43	.011*
Abdominal Pain	.65	.92	3.60	.081

Combined Analyses				
Program Pair	Choice	Matching	$\chi 2(1)$	*p*
Chest Pain	.60	.89	8.074	.005*
Sores	.50	.84	10.47	.001*
Tendonitis	.67	.91	6.60	.010*
Arthralgia	.54	.82	.71	.008*
Onycholysis	.63	.84	4.63	.031*
Eczema	.56	.84	7.49	.006*
Depression	.60	.86	6.55	.010*
Migraine	.48	.82	10.49	.001*
Abdominal Pain	.56	.88	9.95	.002*

Note: Chi-square tests for independence comparing the choice and matching dependent variables for each item failed to reach across all items in Study 1a and 1b, but all were directionally consistent with H1, and reached significance in the combined analyses for Choice and Matching conditions.
