# Supplementary material for: Preference reversals in ethicality judgments of medical treatments
Source: PLoS One. 2025 Apr 29;20(4):e0319233. doi: 10.1371/journal.pone.0319233 (PMC12040148; doi:10.1371/journal.pone.0319233)
Supplement: S6 Table — Study 2 Supplemental Analyses. (PDF) [file pone.0319233.s025.pdf]

Table S6. Proportion of Participants Indicating the Higher-efficacy/symptom-present Program is More Ethical in Study 1 in Rating vs Matching

		**Study 1a**		
Program Pair	Rating	Matching	$\chi 2(1)$	*p*
Chest Pain	.63	.84	1.55	.214
Sores	.58	.79	1.22	.269
Tendonitis	.67	.85	1.18	.277
Arthralgia	.54	.75	1.36	.244
Onycholysis	.61	.80	1.22	.269
Eczema	.63	.75	0.312	.576
Depression	.63	.75	0.312	.576
Migraine	.43	.75	3.68	.055
Abdominal Pain	.60	.84	2.17	.141
		**Study 1b**		
Program Pair	Rating	Matching	$\chi 2(1)$	*p*
Chest Pain	.59	.92	4.79	.029*
Sores	.96	.88	0.22	.634
Tendonitis	.77	.96	1.71	.191
Arthralgia	.47	.88	6.43	.011*
Onycholysis	.64	.88	1.81	.178
Eczema	.71	.92	1.98	.160
Depression	.73	.96	2.41	.121
Migraine	.82	.88	<0.01	.949
Abdominal Pain	.53	.92	6.13	.013*

Combined Analyses				
Program Pair	Rating	Matching	χ2(1)	*p*
Chest Pain	.61	.89	7.33	.007*
Sores	.71	.84	1.34	.248
Tendonitis	.71	.91	4.346	.036*
Arthralgia	.51	.82	8.25	.004*
Onycholysis	.62	.84	4.58	.032*
Eczema	.66	.84	3.17	.075
Depression	.67	.86	3.63	.057
Migraine	.58	.82	5.29	.021*
Abdominal Pain	.57	.88	9.22	.002*

Note: Chi-square tests for independence comparing the choice and rating dependent variables for each item failed to reach across all items in Study 1a and 1b, but were directionally consistent with H1, and reached significance for all but three items in the combined analyses for Rating and Matching conditions.
