# Supplementary material for: Preference reversals in ethicality judgments of medical treatments
Source: PLoS One. 2025 Apr 29;20(4):e0319233. doi: 10.1371/journal.pone.0319233 (PMC12040148; doi:10.1371/journal.pone.0319233)
Supplement: S7 Table — (PDF) [file pone.0319233.s026.pdf]

Table S7. Proportion of Response Type in Study 2 by Item in the Choice and Matching Conditions

Program Pair	Predicted PRs	Opposite PRs	No PR High-Efficacy Preferred	No PR Low-Efficacy Preferred	Total Responses (Ties Excluded)
Chest Pain	21	9	16	18	64
Sores	19	4	15	24	62
Tendonitis	24	13	19	11	67
Arthralgia	15	13	22	12	69
Onycholysis	17	9	22	20	68
Eczema	19	11	21	18	69
Depression	15	10	22	18	65
Migraine	19	8	18	21	66
Abdominal Pain	16	15	22	12	65

Note: Predicted PRs represent the proportion of participant responses in which the higher-efficacy/symptom-present program is preferred in matching, but the lower-efficacy-symptom/absent program is preferred in choice
