# Supplementary material for: Preference reversals in ethicality judgments of medical treatments
Source: PLoS One. 2025 Apr 29;20(4):e0319233. doi: 10.1371/journal.pone.0319233 (PMC12040148; doi:10.1371/journal.pone.0319233)
Supplement: S8 Table — (PDF) [file pone.0319233.s027.pdf]

Table S8. Proportion of Response Type in Study 2 by Item in the Rating and Matching Conditions

Program Pair	Predicted PRs	Opposite PRs	No PR High-Efficacy Preferred	No PR Low-Efficacy Preferred	Total Responses (Ties Excluded)
Chest Pain	18	7	15	13	53
Sores	13	8	10	16	47
Tendonitis	10	6	18	10	44
Arthralgia	11	9	19	7	46
Onycholysis	15	6	15	15	51
Eczema	17	10	13	9	49
Depression	16	6	11	17	50
Migraine	14	8	6	17	45
Abdominal Pain	12	7	15	11	45

Note: Predicted PRs represent the proportion of participant responses in which the higher-efficacy/symptom-present program is preferred in matching, but the lower-efficacy-symptom/absent program is preferred in choice
