# Supplementary material for: Preference reversals in ethicality judgments of medical treatments
Source: PLoS One. 2025 Apr 29;20(4):e0319233. doi: 10.1371/journal.pone.0319233 (PMC12040148; doi:10.1371/journal.pone.0319233)
Supplement: S9 Table — Study 3 Supplemental Results. (PDF) [file pone.0319233.s028.pdf]

Table S9. Correlation Matrix, Study 2, PR Susceptibility Coding Scheme 2

Measure	1	2	3	4	5	6	7	8	9	10	11
1. Susceptibility to Reversals	-	-.004	-.040	-.100	-.157	.006	.045	.005	.195*	.087	-.065
2. BFI-Openness		-	.151	.212*	.147	.280**	-.009	.140	-.019	.010	-.017
3. BFI-Conscientiousness			-	.187*	.399**	.182*	.064	.037	.009	.020	.012
4. BFI-Extraversion				-	.148	.167*	-.095	.098	.034	.173*	-.081
5. BFI-Agreeable					-	.288**	.019	.150	-.013	.062	.078
6. BFI-Neuroticism						-	.184*	.175*	.048	.197*	-.076
7. REI-Rational							-	.354**	.107	.172*	-.090
8. REI-Experiential								-	.111	.181*	.047
9. MIS-Internalization									-	.419**	.054
10. MIS-Symbolization										-	-.045
11. ATS											-

Note: Correlations in the coding scheme in which any PR was coded as 1 and any non PR was coded as 0.

**Indicates $p < .01$ (2-tailed) *Indicates $p < .05$ (2-tailed)
