# Supplementary material for: Preference reversals in ethicality judgments of medical treatments
Source: PLoS One. 2025 Apr 29;20(4):e0319233. doi: 10.1371/journal.pone.0319233 (PMC12040148; doi:10.1371/journal.pone.0319233)
Supplement: S10 Table — (PDF) [file pone.0319233.s029.pdf]

Table S10. Effects of Presentation Mode, Efficacy, and Act/Omission Manipulations on Ratings Before Exclusions

Condition	*df*	*SS*	*MS Error*	*F*	*p*	η_p^2
Mode	1	0.00	3.00	0.01	.912	<.001
Act/Omit	1	6.1	3.00	2.05	.154	.008
Mode X Act/Omit	1	1.4	3.00	0.48	.489	.002
Efficacy	1	18.5	2.06	8.97	.003**	.035
Mode X Efficacy	1	25.3	2.06	12.28	< .001**	.048
Act/Omit X Efficacy	1	2.5	2.06	1.20	.275	.005
Mode X Act/Omit X Efficacy	1	0.2	2.06	0.10	.757	<.001
