# Supplementary material for: Preference reversals in ethicality judgments of medical treatments
Source: PLoS One. 2025 Apr 29;20(4):e0319233. doi: 10.1371/journal.pone.0319233 (PMC12040148; doi:10.1371/journal.pone.0319233)
Supplement: S11 Table — (PDF) [file pone.0319233.s030.pdf]

Table S11. Effects of Presentation Mode, Efficacy, and Act/Omission Manipulations on Ratings After Exclusions

Condition	*df*	*SS*	*MS Error*	*F*	*p*	η_p^2
Mode	1	0.40	3.27	0.13	.723	.001
Act/Omit	1	1.1	3.27	0.34	.003	.559
Mode X Act/Omit	1	0.50	3.27	0.15	.704	.001
Efficacy	1	17.1	1.76	9.70	.002**	.074**
Mode X Efficacy	1	18.3	1.76	10.35	.002**	.078**
Act/Omit X Efficacy	1	0.30	1.76	0.15	.700	.001
Mode X Act/Omit X Efficacy	1	0.70	1.76	0.41	.521	.003
