# Supplementary material for: Preference reversals in ethicality judgments of medical treatments
Source: PLoS One. 2025 Apr 29;20(4):e0319233. doi: 10.1371/journal.pone.0319233 (PMC12040148; doi:10.1371/journal.pone.0319233)
Supplement: S12 Table — (PDF) [file pone.0319233.s031.pdf]

Table S12. Independent Samples t-tests by Program Across Condition Collapsing Across the Action and Omission Manipulation

Study 3										
	**High-Efficacy**					**Low-Efficacy**				
Program	***SE***	***JE***	***t(124)***	***p***	***d***	***SE***	***JE***	***t(124)***	***p***	***d***
Chest Pain	5.74 (2.07)	6.53 (1.83)	-2.15	.033*	-0.38	5.91 (2.12)	5.55 (2.32)	0.89	.375	0.16
Sores	6.16 (1.86)	6.41 (1.97)	-0.73	.469	-0.13	6.14 (2.16)	5.73 (1.93)	1.08	.283	0.19
Tendonitis	6.14 (1.81)	6.86 (1.40)	-2.35	.020*	-0.42	5.99 (2.04)	5.57 (2.20)	1.08	.282	0.19
Arthralgia	6.14 (1.83)	6.69 (1.43)	-1.79	.076	-0.32	6.06 (2.17)	5.55 (2.09)	1.32	.191	0.23
Onycholysis	6.10 (1.80)	6.59 (1.73)	-1.50	.135	-0.27	5.81 (2.13)	5.61 (2.15)	0.49	.622	0.09
Eczema	5.88 (2.03)	6.73 (1.52)	-2.52	.013*	-0.45	5.86 (2.16)	5.27 (2.16)	1.50	.136	0.27
Depression	5.82 (2.16)	6.84 (1.65)	-2.82	.006*	-0.50	5.82 (2.01)	5.10 (2.25)	1.86	.065	0.33
Migraine	5.60 (2.35)	6.41 (1.71)	-2.10	.039*	-0.37	5.84 (1.88)	5.41 (2.22)	1.18	.239	0.21
Abdominal Pain	5.65 (2.10)	6.29 (1.43)	-1.86	.065	-0.33	6.04 (1.98)	5.35 (2.10)	1.87	.064	0.33
